# Small heat shock proteins sequester misfolding proteins in near-native conformation for cellular protection and efficient refolding

**DOI:** 10.1038/ncomms13673

**Published:** 2016-11-30

**Authors:** Sophia Ungelenk, Fatemeh Moayed, Chi-Ting Ho, Tomas Grousl, Annette Scharf, Alireza Mashaghi, Sander Tans, Matthias P. Mayer, Axel Mogk, Bernd Bukau

**Affiliations:** 1 Center for Molecular Biology of the University of Heidelberg (ZMBH), DKFZ-ZMBH Alliance, Im Neuenheimer Feld 282, Heidelberg D-69120, Germany; 2 German Cancer Research Center (DKFZ), Im Neuenheimer Feld 280, Heidelberg D-69120, Germany; 3 FOM Institute for Atomic and Molecular Physics (AMOLF), Science Park 104, Amsterdam 1098 XG, The Netherlands

## Abstract

Small heat shock proteins (sHsp) constitute an evolutionary conserved yet diverse family of chaperones acting as first line of defence against proteotoxic stress. sHsps coaggregate with misfolded proteins but the molecular basis and functional implications of these interactions, as well as potential sHsp specific differences, are poorly explored. In a comparative analysis of the two yeast sHsps, Hsp26 and Hsp42, we show *in vitro* that model substrates retain near-native state and are kept physically separated when complexed with either sHsp, while being completely unfolded when aggregated without sHsps. Hsp42 acts as aggregase to promote protein aggregation and specifically ensures cellular fitness during heat stress. Hsp26 in contrast lacks aggregase function but is superior in facilitating Hsp70/Hsp100-dependent post-stress refolding. Our findings indicate the sHsps of a cell functionally diversify in stress defence, but share the working principle to promote sequestration of misfolding proteins for storage in native-like conformation.

## Abstract (web summary)

Small heat shock proteins (sHsps) contribute to cellular recovery and survival following stress causing elevated levels of misfolded or unfolded proteins. Here the authors demonstrate that sHsps function by maintaining aggregating proteins in close-to-native conformations to facilitate chaperone-mediated refolding.

## Graphical abstract

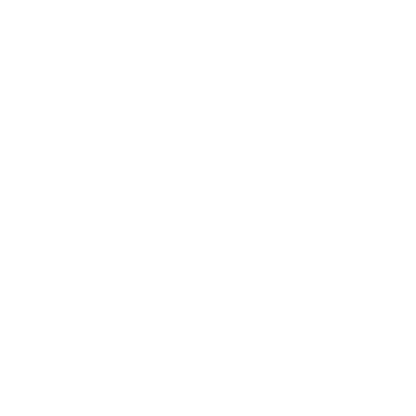

A broad spectrum of stress conditions and physiological imbalances promote protein misfolding, disrupting cellular proteostasis. Misfolded proteins typically expose hydrophobic patches, which target them to the cellular refolding and degradation machineries or nucleate intermolecular aggregation. Under severe stress conditions or during ageing the concentration of misfolded protein may exceed the capacity of the refolding and degradation machineries leading to increased aggregation^1^,^2^,^3^,^4^. Protein aggregates can be cytotoxic and correlate to pathophysiological states including neurodegeneration^5^. Aggregation however, also sequesters potentially toxic protein species and therefore is suggested to have cytoprotective functions^2^,^6^,^7^. A protective function is in line with recent findings that aggregation is an organized process *in vivo* involving dedicated machineries, rather than a stochastic process solely driven by intrinsic physicochemical properties and concentrations of misfolding proteins^8^,^9^.

Small heat shock proteins (sHsps) are evolutionary conserved components of protein quality control networks and constitute a first line of stress defence. sHsp dysfunction is linked to pathological disorders, including cataracts, myopathies and neurodegenerative diseases^10^,^11^. sHsps were initially described to prevent the aggregation of misfolded proteins as ‘holdases'^12^. When present at stoichiometric concentrations, sHsps lead to the formation of large, yet soluble sHsp/substrate complexes^13^,^14^,^15^,^16^. *In vivo*, however, sHsps typically coaggregate with misfolded proteins and become part of insoluble aggregates^17^,^18^,^19^,^20^. sHsp activity can even be required for the formation of microscopically visible protein aggregates. Thus in *S. cerevisiae*, the sHsp Hsp42 triggers cytosolic CytoQ aggregate formation on stress (also referred to as Q-bodies) and is integral part of these^21^,^22^,^23^. How these seemingly opposing activities of sHsps in both, prevention and formation of protein aggregates are related is unclear. The failure so far to reconstitute an ‘aggregase' activity for sHsps *in vitro* led to the assumption that additional factors are involved in promoting aggregation inside cells.

sHsp–substrate complexes are usually stable, requiring the action of ATP-dependent Hsp70 and Hsp100 chaperones for substrate release and subsequent refolding^15^,^16^,^24^,^25^,^26^. Refolding of substrates from complexes with sHsps occurs with higher efficiency and faster kinetics as compared with substrates aggregated without sHsps, which is critically important for cellular stress recovery and survival^20^,^27^. However, the molecular basis for this activity is poorly understood. In particular, we lack information on the molecular features of stress-induced aggregates of misfolded proteins, and the effects of sHsps on these aggregates. Moreover, the number of sHsp family members expressed in cells increased during evolution, from 1 to 2 members in bacteria and yeast to 10 members in human and >25 members in plant cells^28^, but the extent by which sHsp family members differ in chaperone activity and physiological roles is unclear. Initial evidence for functional diversification of bacterial and plant sHsps activities has been provided *in vitro*^29^,^30^,^31^. sHsp family members are furthermore differentially activated. *S. cerevisiae* Hsp26 requires heat shock temperatures for activation, while the second sHsp of yeast, Hsp42, is constitutively active^32^,^33^,^34^.

Here, we investigate the mode of action and potential functional diversification of a cell's set of sHsps using *S. cerevisiae* Hsp26 and Hsp42 as models. Using single molecule and hydrogen exchange experiments, we determine the conformational state of aggregates of mechanically or thermally denatured proteins formed in the absence or presence of Hsp42 and Hsp26. Heat-aggregated proteins are globally unfolded, but on association with either Hsp42 or Hsp26 become stabilized in near-native conformation. Both sHsps thus capture proteins early during stress-induced unfolding. Hsp42, but not Hsp26, actively promotes formation of large sHsp–substrate assemblies *in vitro* under mild denaturing conditions. The reconstitution of an aggregase function of Hsp42 demonstrates that this sHsp is sufficient for promoting protein aggregation without additional cellular factors. The diversification in sHsp activities that we identify *in vitro* is correlated to distinct *in vivo* phenotypes of *hsp42*Δ and *hsp26*Δ mutant cells. We show that cellular fitness during repetitive stress treatments specifically requires Hsp42 activity, demonstrating that organized protein aggregation is cytoprotective.

## Results

### Single-molecule analysis of sHsp activity

**Figure 1:**
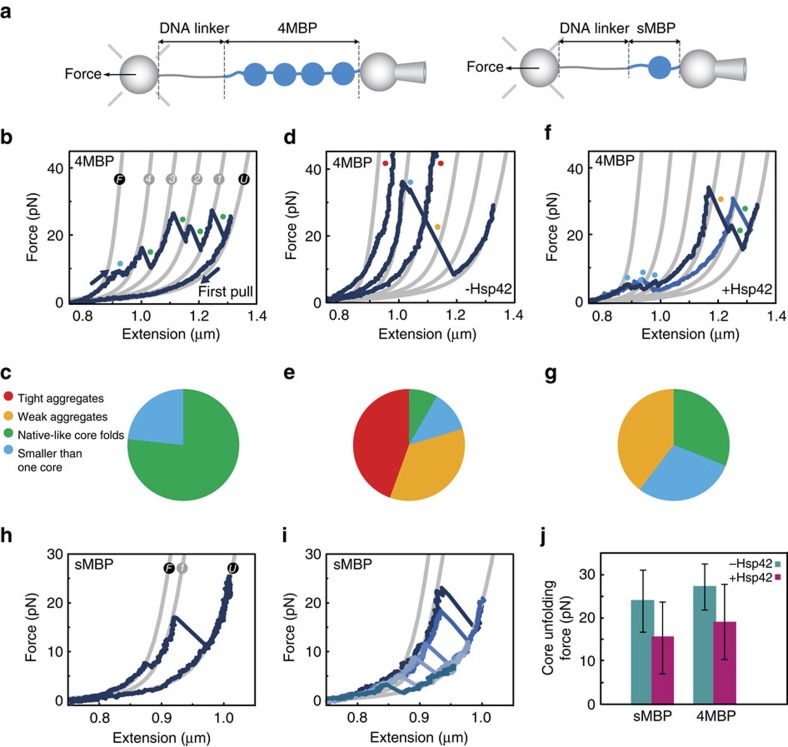
Effect of Hsp42 at the single-molecule level. (**a**) Schematic diagram of the setup; MBP constructs are tethered by means of a DNA handle between two beads: one held on a position-controlled micropipette, another by an optical trap that allows force detection. (**b**) First stretching–relaxation curve, showing the unfolding pattern of natively folded 4MBP. Grey lines represent the theoretical WLC characterizing the DNA-4MBP construct from fully folded (F) to the fully unfolded (U) state. After C-terminal unfolding (F→4) in all four MBP repeats, four core unfolding events (4→3→2→1→U) are observed. Coloured points indicate the type of observed protein structures (Supplementary Fig. 1). The category ‘smaller than one core' includes gradual transitions, as seen here for F→4. (**c**) Corresponding fractions of the types of observed structures (*N*=20). Fractions are normalized by the length of the amino acid chains that form the structure. (**d**) Second or subsequent stretching curves after relaxation and waiting at zero force for 5 s without chaperone. (**e**) Corresponding fractions of the types of observed structures (*N*=30). (**f**) Second or subsequent stretching curves in the presence of Hsp42 (5 μM). (**g**) Corresponding fractions of the types of observed structures (*N*=30). (**h**) Stretching curves for sMBP. Grey lines represent WLC model for DNA-sMBP for the fully folded (F), the single core (1) and fully unfolded (U) states. (**i**) Stretching curves in the presence of Hsp42 (5 μM). (**j**) Corresponding core unfolding forces (*N*=51 and *N*=42 for sMBP and 4MBP respectively).

To determine the effects of sHsps on substrate molecules at the most reductionist level, we performed single-molecule unfolding-aggregation assays. We used optical tweezers to stretch a single polyprotein composed of four repeats of maltose binding protein (4MBP) mimicking a high local protein concentration (Fig. 1a). As shown previously^35^, stretching individual 4MBP molecules for the first time results in a gradual unfolding transition (Fig. 1b,c, F→4), corresponding to the unfolding of four C-terminal α-helixes in one go, followed by distinct unfolding of the four remaining core structures (4→3→2→1→U). In each of these unfolding events, the measured tether length suddenly increases by about 92 nm, which corresponds to the peptide-length of one core structure, at a mean force of about 25 pN (Supplementary Fig. 1). In the absence of chaperone, after relaxation to low force, subsequent stretching curves (Fig. 1d,e) often show compact structures that resist unfolding (unfolding force higher than 65 pN), and were termed tight aggregates (Supplementary Fig. 1). We also observed weaker non-native structures that unfold above 35 pN and/or released chain segments exceeding one core structure, which we here refer to as weak aggregates (Supplementary Fig. 1). Note that because of the physical linkages between MBP repeats, the geometry of the small aggregates studied here should be different than aggregates of MBP monomers. Altogether, we find that isolated polypeptide chains predominantly form misfolded and aggregated structures involving more than one repeat.

Next, similar single-molecule assays were performed in the presence of Hsp42, which is constitutively active. Our experimental setup did not allow testing Hsp26, as it requires increased temperature for activation. Stretching curves for the first 4MBP unfolding are similar irrespective of the presence or absence of Hsp42. This indicates that Hsp42 does not interact with natively folded repeats. On complete unfolding and relaxation to low force in the presence of Hsp42, subsequent stretching curves show important differences (Fig. 1f,g). First, tight aggregates are now absent (Fig. 1g). This shows Hsp42 suppresses non-native intra-molecular contacts between protein domain repeats. Weak aggregates are still observed (Fig. 1f,g), indicating suppression is not perfect, possibly because client monomers have a very high effective local concentration in this assay. Also, there is substantial increase in the number of folded core structures that unfold in native-like manner (Fig. 1f,g). In these cases, the measured tether length increased by about 92 nm at forces below 35 pN. Interactions with Hsp42 therefore promote the formation of native-like structures.

These results give rise to further questions regarding the nature of the Hsp42–substrate complex. In particular, we also observed an increased frequency of unfolding events with small length increases (smaller than one core, Fig. 1f,g). This could either indicate the detachment of a peptide segment from a larger aggregated structure, or a small native-like independent structure complexed with Hsp42, as seen previously for trigger factor^36^. To clarify this, we focused on a single-repeat MBP protein (sMBP), which can refold but cannot aggregate, as there are no aggregation partners. In stretching–relaxation assays in the absence of chaperone (Fig. 1h), sMBP either fully refolds and then unfolds in native-like manner, or does not refold at all, and does not show further unfolding features during subsequent stretching. These data reconfirm that sMBP spontaneously refolds, without the assistance of chaperones^35^. In the presence of Hsp42, stretching assays on sMBP show that unfolding steps smaller than one core are absent (Fig. 1i), in contrast to the 4MBP data with Hsp42 (Fig. 1f,g). Rather, the unfolded and relaxed chains either did not refold at all or refolded to a core-like state, which unfolded in a single-step of 92 nm (Fig. 1i). This behaviour indicates that the small length changes seen for 4MBP originate from (partial) disruption of aggregated states.

Interestingly, we found that the force required to unfold the sMBP refolded core-like states was lower with Hsp42 present than without (15 pN versus 24 pN, *P*<0.05, Fig. 1j). This decrease could potentially indicate refolding into other non-native MBP states unbound by Hsp42 that were less resistant against forced unfolding. However, this scenario would require that Hsp42 altered the refolding pathway in a specific and reproducible way and then dissociated. Moreover, these MBP states would need to be inaccessible without Hsp42, unfold in a similar single-step of about 92 nm, be less force-resistant than the native core state, but still stable enough to be observed in the assay. Alternatively, Hsp42 presence could lead to near-native MBP core states that remain bound by Hsp42. For instance, by interfering with native intra-molecular contacts in sMBP, Hsp42 could prevent conversion to the fully native core state resulting in a lower resistance against forced unfolding. Consistently, we find that a similar decreased unfolding force is observed for refolded cores in the 4MBP construct in the presence of Hsp42 (Fig. 1j). To further probe interaction with the extended state, we kept unfolded sMBP at 5 pN for 15 s before refolding at 0 pN. This increased exposure time did not significantly affect the refolding rate and again led to refolded cores with a lower unfolding force of 15 pN (*P*<0.05, *N*=17). These data are consistent with a limited effect of Hsp42 before MBP refolding.

We note that we did not find evidence for Hsp42 binding to fully native MBP, as indicated by the first stretching curve on sMBP or 4MBP in the presence of Hsp42 that showed native unfolding (Fig. 1b). Overall, these data suggest that Hsp42 can promote and interact with near-native MBP structures, while suppressing their aggregation.

### Heat-aggregated MDH is globally unfolded

**Figure 2:**
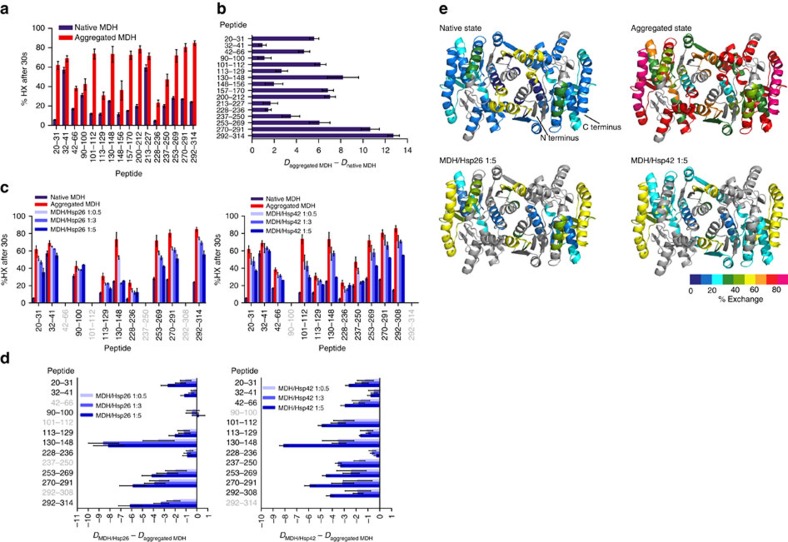
Hsp26 and Hsp42 protect unfolded regions of heat-aggregated MDH from HX. (**a**) Relative proton/deuteron exchange in native and heat-aggregated MDH after 30 s incubation in D_2_O. (**b**) Difference in deuteron incorporation of peptic peptides between native and aggregated MDH. (**c**) Deuteron incorporation into MDH co-aggregated with either Hsp26 or Hsp42 was determined after 30 s labelling. sHsp/MDH complexes were performed by incubation at 47 °C for 30 min. (**d**) Difference in deuteron incorporation between heat-induced MDH/Hsp26 (left) and MDH/Hsp42 (right) complexes and aggregated MDH. Peptides in grey could not be identified. Error bars denote s.d. for each point based on three repetitions. All data were corrected for deuteron losses due to back-exchange using a 100% deuterated control. (**e**) HX-heat map of native, aggregated and sHsp-complexed states of the MDH dimer structure (PDB ID 1MLD). Peptic peptides are coloured according to their exchange behaviour (per cent exchange). Grey regions could not be detected. The ratio of sHsp versus MDH during substrate denaturation is given.

sHsps typically form multimeric complexes with aggregation-prone proteins, each of which comprising multiple sHsp and substrate molecules^16^,^37^,^38^. It is therefore possible that the conformational state of substrates in these complexes is different to that of isolated substrate molecules observed at the single-molecule level. To assess the effects of sHsp binding on substrate conformation within these multimeric complexes, we first determined the structure of an aggregating thermolabile model substrate, malate dehydrogenase (MDH)^26^, subjected to heat-induced aggregation at 47 °C in the absence of sHsps. The conformational changes in MDH were analysed by amide hydrogen exchange (HX) mass spectrometry (MS), which determines the solvent accessibility and structural flexibility of the peptide backbone. Amide hydrogens are protected from HX if engaged through hydrogen bonds in secondary and tertiary structures or protein–protein interactions. We compared the HX pattern of peptic peptides from native versus heat-aggregated MDH (Fig. 2). Pepsin degrades at least 50% of MDH aggregates and no major degradation products are detectable, showing most aggregated MDH species can be analysed by HX at the peptide level (Supplementary Fig. 2a). We achieved high sequence coverage for native (83%) and aggregated (76%) MDH. Some variability exists between individual HX measurements for aggregated MDH (10–20% deviation of total HX for individual peptides), but a consistent trend is observed between assays for all peptides.

Aggregation of MDH leads to profound loss of organized structure, reflected in increased HX for all identified peptides distributed throughout the MDH sequence and structure (Fig. 2a,b,e; Supplementary Fig. 2b). Many peptides show between 60 and 80% exchange indicating extensive unfolding and solvent exposure. Peptic peptides that show minor HX increases (Tyr32–Ala41, Val213–Met227) rapidly exchange already in the native state. In the aggregated state, peptide Ala228–Phe236, which forms an α-helix at the dimer interface of native MDH and via Tyr229 interacts directly with Glu48 of peptide 42–66 of the second protomer, maintained the lowest HX (Fig. 2e; Supplementary Fig. 2c). Peptide 42–66 shows intermediate exchange in the aggregated state (Fig. 2a,b). Another peptide located at the dimer interface however, exchanges rapidly in the aggregated state (Val157–Val170), arguing against a complete MDH dimer core structure remaining in the aggregate (Supplementary Fig. 2c). Altogether, the HX assay results indicate that MDH aggregates consist of globally misfolded conformers lacking substantial secondary and tertiary structures in many regions of the protein.

### sHsp binding globally protects heat-denatured MDH from HX

We next assessed the structural changes of MDH resulting from the association with Hsp42 or Hsp26. These sHsps are equally efficient in preventing formation of large MDH aggregates at 47 °C as tested by turbidity and solubility assays (Supplementary Fig. 3a,b). Varying sHsp levels during substrate denaturation produced sHsp/MDH complexes of different sizes. Substoichiometric levels of Hsp26 or Hsp42 relative to MDH yielded large, insoluble sHsp/MDH complexes, whereas molar excess of the chaperones resulted in smaller soluble complexes (Supplementary Fig. 3a,b). At all conditions, MDH bound to sHsps remained inactive, but compared with MDH aggregates formed in the absence of sHsps, excess sHsp supported faster and more efficient MDH reactivation by the yeast bi-chaperone disaggregase system formed by Ssa1 (Hsp70), Sis1 (Hsp40), Sse1 (Hsp110) and Hsp104 (Hsp100) (Supplementary Fig. 3c). Earlier reports for a range of substrates agree with these observations^18^,^24^.

HX assays on heat-induced sHsp/MDH complexes generated in presence of low or high sHsp versus MDH ratios, allowed us to determine the complete structural spectrum of the complexes (Fig. 2c,d). We identified a smaller number of peptic MDH peptides from sHsp/MDH complexes compared with that for aggregated MDH. Reduced recovery is not due to inefficient pepsin digest (Supplementary Fig. 2a) and stems from interference by sHsp peptic peptides due to overlapping mass spectra. Analysis of the mass spectra of MDH peptides revealed bimodal peak distributions, indicating distinct MDH peptide conformations (discussed below). We first calculated the overall degree of HX protection of MDH peptides on sHsp binding by calculating the average centroid over both populations.

Increasing concentrations of both sHsps continuously increased HX protection of most identified MDH peptides to a similar degree (Fig. 2c,d,e). Hsp42 and Hsp26 both exerted a global effect, protecting MDH peptides dispersed throughout sequence and structure. HX was still higher as compared with native MDH, suggesting that sHsp-bound MDH has retained significant structural flexibility (Fig. 2e; Supplementary Fig. 4). The peptide Val130–Leu148, largely buried inside the hydrophobic core of native MDH (Supplementary Fig. 6c), showed strongest protection on sHsp complex formation, yielding a native-like HX pattern (Fig. 2d; Supplementary Fig. 4). Here, Hsp26 was more efficient compared with Hsp42, conferring strong protection to this peptide if present at threefold excess, while fivefold excess of Hsp42 was needed to yield comparable HX. Such difference was not apparent in the less sensitive turbidity and solubility assays, demonstrating that HX can report on differences in sHsp–substrate interactions not detectable by conventional chaperone assays.

### Hsp26 and Hsp42 stabilize MDH in a native-like state

**Figure 3:**
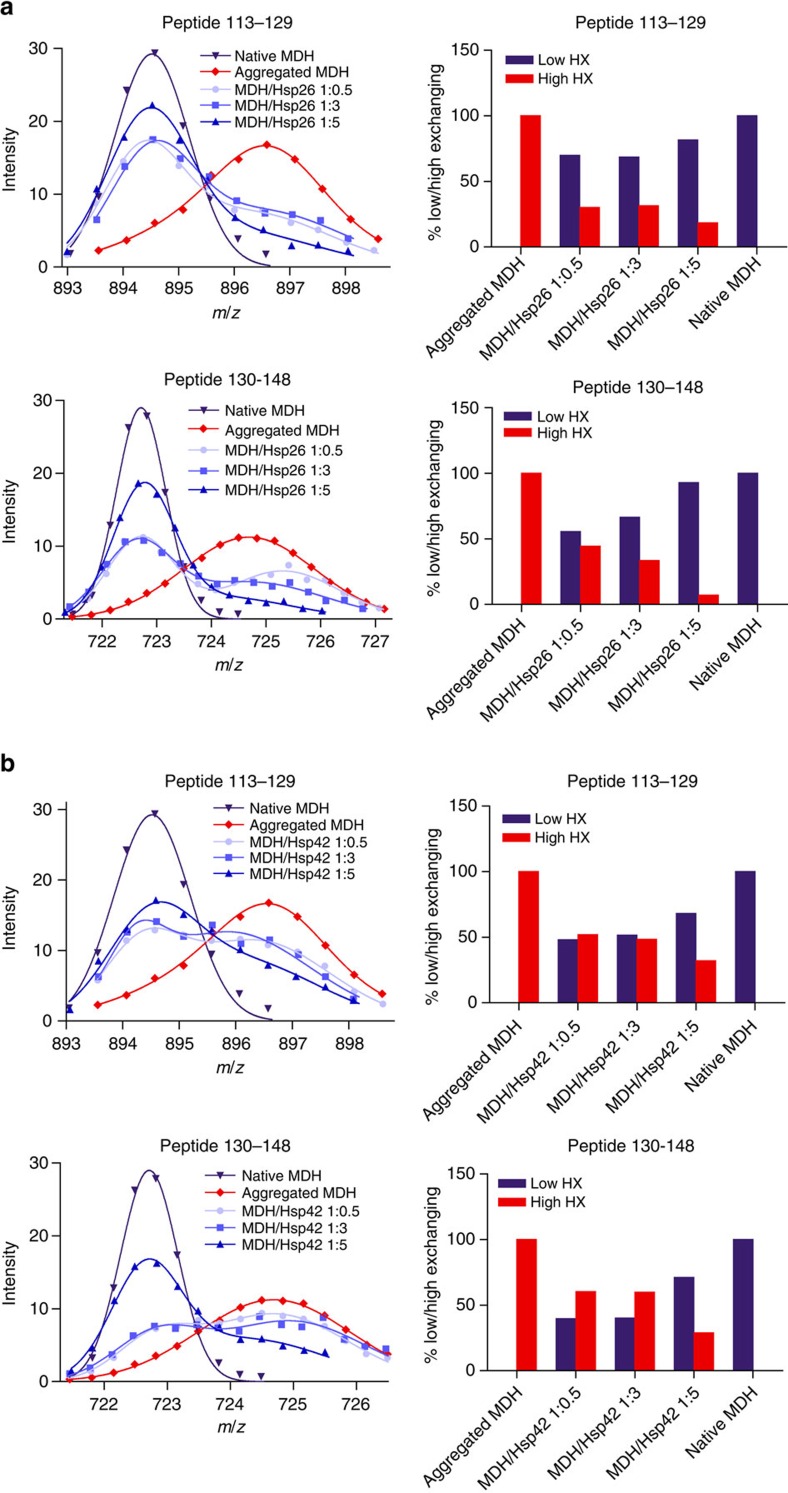
sHsps stabilize segments of bound MDH in a native-like state. Bimodal distribution of isotope peaks of indicated MDH peptides derived from MDH/Hsp26 (**a**) and MDH/Hsp42 (**b**) complexes. Left panels: Intensity versus *m*/*z* diagrams for different peptic MDH fragments after 30 s HX at 30 °C. Right panels: Fractions of native-like and aggregate-like populations calculated for respective peptides.

HX analysis reveals a bimodal peak distribution of most peptic MDH peptides derived from complexes with either Hsp26 or Hsp42 (Fig. 3; Supplementary Figs 5 and 6). Such a bimodal distribution reflects the coexistence of two structural states: a low-exchanging and a high-exchanging population, peak positions of which were similar to the peak positions of the same peptide derived from native or aggregated MDH (native-like and aggregate-like). Bimodal distributions were not observed for native MDH and only to a minor extent for few peptides of aggregated MDH (Leu20–Leu31, Ala42–His46). Increasing the sHsp concentration relative to MDH during substrate denaturation leads to a shift of the population distributions from aggregate-like to native-like. Native-like HX patterns of MDH peptides can therefore be attributed to efficient sHsp/MDH complex formation. MDH peptides Val130–Leu148 (when associated with Hsp26 or Hsp42) and Ile113–Glu129 (when associated with Hsp26) exhibited almost exclusively native-like states. Notably, these peptides are largely hidden in the interior of native MDH, suggesting that MDH retains substantial tertiary structure in complex with sHsps (Supplementary Fig. 6c). This trend occurs to a varying degree for all identified MDH peptides and for both, Hsp26 and Hsp42. However, we noticed that Hsp26 effects were stronger for most peptides in particular at lower sHsp levels (Supplementary Fig. 6a,b).

Native-like HX patterns of peptic MDH peptides could arise from partial MDH refolding after spontaneous release from sHsp/MDH complexes. To exclude this possibility, we tested the stability of sHsp/MDH complexes by adding ATPase-deficient GroEL-D87K, which traps misfolded proteins including MDH^20^,^39^. Incubation of Hsp26/MDH or Hsp42/MDH complexes with GroEL-trap did not result in MDH transfer to GroEL (Supplementary Fig. 7a). Along the same line, free MDH was not detected but was found in complex with either sHsps (Supplementary Fig. 7b). This finding excludes spontaneous dissociation of non-native MDH from sHsp/MDH complexes and demonstrates that the native-like MDH peptide conformations defined experimentally represent sHsp-bound but not free MDH.

### Mapping of sHsp–MDH interaction sites

**Figure 4:**
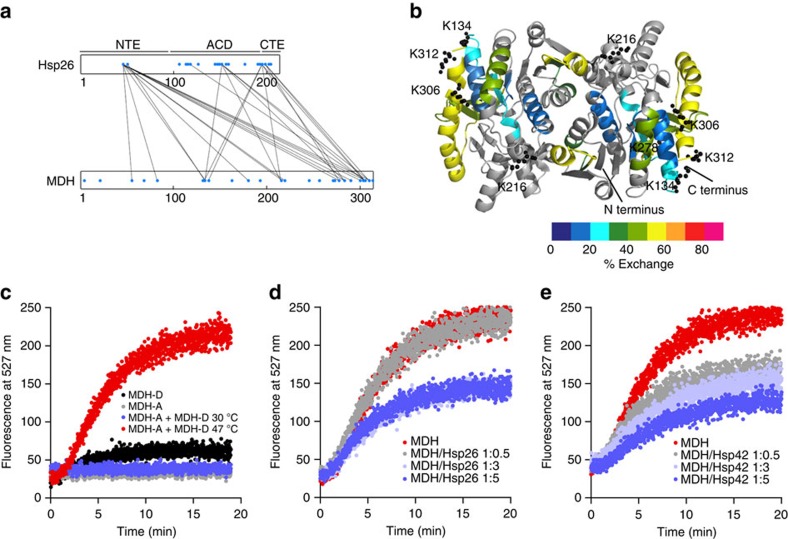
Specific interactions of sHsps with MDH cause separation of sequestered MDH molecules. (**a**) Specific interactions of Hsp26 with exposed, flexible MDH segments. MDH–Hsp26 interaction sites were determined by DSS crosslinking and mass spectrometry analysis. Linear representation of crosslinks between lysine residues (blue dots) of Hsp26 and MDH. (**b**) HX-heat map of MDH from the heat-induced MDH/Hsp26 (1:5 ratio) complex. Peptic peptides are coloured according to their exchange behaviour (per cent exchange). Grey regions could not be identified. Lysine residues of MDH that were crosslinked to two or more lysines of Hsp26 are presented as balls and sticks. (**c**) Co-aggregation of MDH–YFP (FRET donor) and MDH labelled with 7-diethylcoumarin-3-carboxylic acid (FRET acceptor) causes specific FRET increase at 47 °C. Mixture of FRET donor and acceptor at 30 °C and acceptor or donor only controls at 47 °C do not result in FRET. Presence of Hsp26 (**d**) or Hsp42 (**e**) reduces FRET efficiencies between MDH–YFP and 7-diethylcoumarin-3-carboxylic acid-labelled MDH in a concentration-dependent manner.

To determine the identity of substrate segments directly captured by sHsps on unfolding, and to identify regions within Hsp26 and Hsp42 involved in substrate binding, we performed chemical (DSS) crosslinking of sHsp/MDH complexes and identified sHsp–MDH crosslink products by mass spectrometry (Fig. 4a; Supplementary Fig. 8; Supplementary Table 1). DSS crosslinks lysine residues located within a Cα–Cα distance of <30 Å(ref. 40).

We identified multiple Hsp26–MDH crosslinks, which involve a subset of theoretically available lysine residues in MDH and Hsp26, demonstrating specificity (Fig. 4a; Supplementary Table 1). In MDH, Hsp26 crosslinked to five lysines (Lys134, Lys216, Lys278, Lys306, Lys312) of which three (Lys134, Lys306, Lys312) cluster in close proximity in the structure of native MDH. All crosslinked lysines are present in peptide segments of MDH that showed strong deprotection in the aggregated state. HX of these peptides was strongly decreased on sHsp binding, but still stayed high compared with other MDH peptides (Fig. 4b). This holds true in particular for the exposed C-terminal α-helix (Ile292–Met314), which includes two major Hsp26 crosslink sites (Fig. 4b). In Hsp26, lysine residues of the N-terminal extension (NTE), the conserved α-crystallin domain (ACD) and the C-terminal extension (CTE) crosslinked to MDH (Fig. 4a; Supplementary Table 1). The frequency of crosslinks differed strongly between Hsp26 domains. All lysine residues (two) of the NTE were crosslinked to substrate compared with two out of twelve and two out of six lysines of the ACD and CTE, respectively. ACD–MDH crosslinks were only detected at higher Hsp26 concentrations, suggesting that the ACD offers additional low-affinity substrate binding sites. Lys45 of the NTE formed the highest number of crosslink products to MDH. Notably, Lys45 is part of the central thermosensor region of the Hsp26 NTE, which changes conformation on heat activation. Other major Hsp26 crosslink sites to substrates include Lys151 of ACD (loop connecting β5 and β7) and Lys195/Lys198 of CTE (Fig. 4a; Supplementary Table 1).

We failed to identify DSS-crosslinked lysines in Hsp42–MDH complexes, although Hsp42/MDH complex formation was confirmed by determining an altered MDH crosslink pattern in western blot analysis (Supplementary Fig. 8b). We notice that the N-terminal 171 residues of Hsp42 do not include lysine residues and are therefore not accessible to DSS crosslinking. An Hsp42 deletion variant lacking the N-terminal region (residues 1–243) is deficient in promoting CytoQ formation *in vivo*^21^, implying that this region is involved in substrate interaction but cannot be detected by our crosslinking approach.

To corroborate these results regarding the substrate interacting segments of Hsp26 and Hsp42, we determined HX profiles of free Hsp26 and Hsp42 and their complexes with denatured MDH. For both sHsps HX was highest in the NTEs, indicating high flexibility of these regions (Supplementary Fig. 9a,b). On substrate binding, changes in HX profiles were noticed for both sHsps. In Hsp26 two peptides (Phe11–Phe21, Val132–Leu140) became deprotected, indicating increased flexibility in the complexed state (Supplementary Fig. 9c). Increased HX protection was determined for Tyr33–Leu62 in the NTE thermosensor region of Hsp26, encompassing Lys45 identified as main DSS crosslinking site to bound MDH. In Hsp42 binding to MDH caused increased HX protection in all identified peptides belonging to NTE (Supplementary Fig. 9d). Together these findings support a function of flexible NTEs as major substrate binding sites of Hsp26 and Hsp42, consistent with previous reports for various sHsps from other species^41^,^42^,^43^.

### sHsp association separates sequestered MDH molecules

The ability of Hsp42 and Hsp26 to store misfolded proteins in native-like state raises the question of how bound molecules are prevented from forming tight aggregates with each other. We therefore investigated the global architecture of sHsp–substrate complexes with the aim to elucidate the spatial relationship of the trapped substrate molecules relative to each other. We assessed fluorescence resonance energy transfer (FRET) between the FRET pair MDH–YFP and MDH labelled with 7-diethylcoumarin-3-carboxylic acid. FRET was observed at 47 °C but not at 30 °C, confirming that FRET reports on co-aggregation of MDH donor and acceptor molecules (Fig. 4c). On heat shock, the FRET signal increases much faster than sample turbidity (Supplementary Fig. 3a), indicating a higher sensitivity for this assay. The presence of Hsp26 or Hsp42 reduces FRET efficiencies in a concentration-dependent manner (Fig. 4d,e). This indicates that increasing sHsp binding increases the spacing between MDH molecules and prevents intermolecular contacts of misfolded MDH species.

### Hsp42 but not Hsp26 promotes MDH aggregation

Evidence provided so far shows that both sHsps from yeast, Hsp26 and Hsp42, share the abilities to stabilize bound substrates in near-native conformation and facilitate subsequent refolding by Hsp70/Hsp100. *In vivo* however, Hsp42 and Hsp26 exhibit substantial differences in cellular function^32^,^33^. Hsp42 in particular promotes the formation of large, microscopically traceable aggregates during moderate physiological stress conditions (37 °C)^21^. This function of Hsp42 is in striking discrepancy to its activity *in vitro* where Hsp42 protects MDH from aggregation at 47 °C (Supplementary Fig. 3a,b), similar to the ‘holdase' function reported for other sHsp family members^10^. This discrepancy led to the suggestion that additional cellular factors are involved in aggregate formation *in vivo*. Alternatively, however, we considered that this discrepancy results from the *in vitro* assay conditions, which may mask any aggregation promoting activity of Hsp42. In particular, the applied MDH denaturation conditions (47 °C) cause fast and global unfolding of MDH (Fig. 2), thereby perhaps superseding the need for Hsp42 to trigger aggregation.

**Figure 5:**
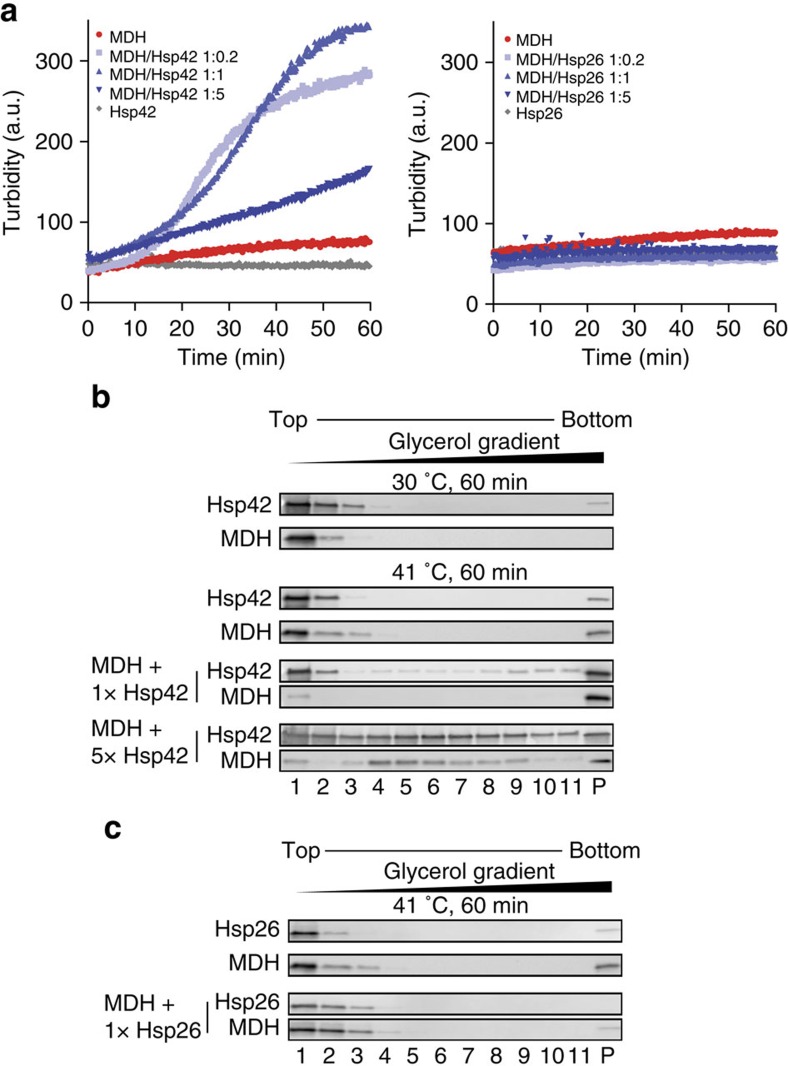
Hsp42 promotes MDH aggregation under mild denaturation conditions. (**a**) MDH (0.5 μM) was denatured for 60 min at 41 °C in the absence or presence of sHsps at various ratios (0.1–2.5 μM). As control 2.5 μM sHsps were heated alone. The formation of turbid MDH aggregates was followed at 600 nm. (**b**,**c**) Samples were prepared as described above, loaded onto a 10–50% glycerol gradient and ultracentrifuged at 40,000 r.p.m. for 1 h at 4 °C. Fractions were analysed by SDS–PAGE followed by western blot analysis (see also Supplementary Fig. 12 for uncropped blots).

We therefore set out to identify a temperature resulting in slower and less global MDH unfolding. We recorded a MDH melting curve using the dye SYPRO orange that exhibits increased fluorescence on binding to hydrophobic protein segments^44^ (Supplementary Fig. 10a). The melting curve confirms that 47 °C represents a harsh treatment as this temperature is close to *T*_M_ for MDH (50.9 °C). Increase in SYPRO orange fluorescence started at 41 °C indicating initiation of MDH unfolding. Consistently, MDH became inactive on incubation at 41 °C, yet inactivation was slower as compared with 47 °C (Supplementary Fig. 10b). On incubation at 41 °C for 60 min, MDH remained largely not-turbid in light scattering measurements and, consistently, the majority of MDH species stayed soluble as shown by glycerol gradient analysis and sample centrifugation (Fig. 5a–c; Supplementary Fig. 10c). In contrast, the addition of substoichiometric (1:0.2) and stoichiometric (1:1) substrate:Hsp42 concentrations triggered MDH aggregation and caused almost quantitative formation of turbid, insoluble MDH aggregates (Fig. 5a,b; Supplementary Fig. 10c). Stimulation of MDH aggregation by Hsp42 relied on direct physical interactions as Hsp42 became part of MDH aggregates (Fig. 5b; Supplementary Fig. 9c). Hsp42 did not accelerate MDH inactivation (Supplementary Fig. 10b) excluding that Hsp42 functions by promoting MDH unfolding. Addition of an excess of Hsp42 reverted the stimulatory effect on MDH aggregation and led to the formation of smaller, non-turbid and more soluble MDH/Hsp42 complexes (Fig. 5a,b; Supplementary Fig. 10c). These findings indicate that both, unfolding conditions and ratio between Hsp42 and substrate are key parameters to control the size of Hsp42/substrate complexes. In contrast, Hsp26 did not promote MDH aggregation at all ratios tested, but exhibited classical ‘holdase' activity by forming non-turbid, soluble complexes with non-native MDH (Fig. 5a,c; Supplementary Fig. 10c).

We then analysed whether promoting or suppressing MDH aggregation at 41 °C by Hsp42 and Hsp26 impact on subsequent MDH reactivation by Hsp70/Hsp100. MDH was refolded with similar kinetics and yields regardless of its aggregation status, indicating that Hsp42-driven MDH aggregation is not detrimental for substrate reactivation (Supplementary Fig. 10d).

### Hsp26 and Hsp42 have distinct cellular functions

Our findings that Hsp26 and Hsp42 have common but also differing activities *in vitro* raise the question whether both sHsps fulfil distinct or redundant functions in cellular protein quality control. However, neither *hsp42*Δ nor *hsp26*Δ mutant cells exhibit obvious growth phenotypes (monitored by colony plating assays) even when exposed to a variety of different stress conditions^24^,^33^. This observation is difficult to interpret since there might be overlapping sHsp functions, compensatory activities of other protein quality control components, or limitations of the plating assays largely reporting on life-death decisions.

**Figure 6:**
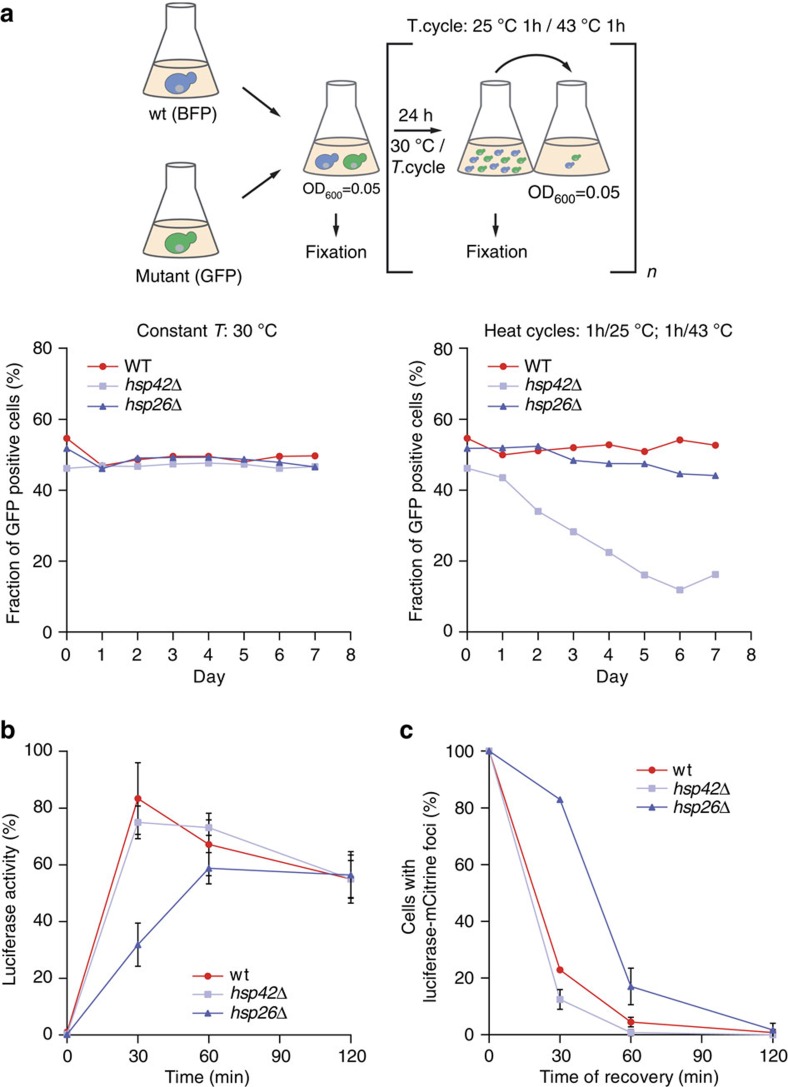
Hsp26 accelerates protein recovery from aggregates and Hsp42 is beneficial for cell survival. (**a**) WT cells, expressing BFP, and respective mutant cells, expressing GFP, were grown to mid-log phase, mixed 1:1 and diluted to OD_600_ 0.05. The mixed culture was equally split and both were grown for seven days with daily dilutions to OD_600_ 0.05. One culture was grown at 30 °C (left), the other culture was subjected to temperature cycles switching between 1 h at 25 °C and 1 h at 43 °C (right). Each day the proportion of WT and mutant cells was measured by monitoring the fraction of GFP- and BFP-positive cells by FACS analysis. (**b**) Effect of Hsp26 on luciferase recovery. Cells expressing yEMCitrine-luciferase in the respective genetic backgrounds were subjected to heat shock and allowed to recover. The graph shows the luciferase activity during recovery at 30 °C. Cycloheximide was added before the heat shock. Mean and s.d. of three independent experiments are shown. (**c**) Heat-induced luciferase aggregates are dissolved more slowly in Δ*hsp26* strain than in wild type or Δ*hsp42* strains. Quantification is based on microscopy experiment (Supplementary Fig. 10b–d) and on analysis of ∼100 cells per strain. After heat application (pre-shock for 45 min at 37 °C and subsequent heat shock for 20 min at 45 °C) cells recovered at 30 °C. Relative ratios of cells with luciferase foci during recovery in respective strain backgrounds are shown. s.d. is given.

To increase the sensitivity in phenotypic readout we performed growth competition assays between wild type and *hsp42Δ* or *hsp26Δ* mutant cells. Mixed cultures were kept either at constant growth conditions (30 °C) or subjected to repetitive heat stress cycles (25–43 °C) for 1 week including daily dilutions of cells into fresh media. Temperature up- and downshifts were done in an incubator resulting in more physiological gradual, rather than abrupt temperature changes. Expression of blue fluorescent protein (BFP) and green fluorescent protein (GFP) in wt and sHsp mutant cells, respectively, allowed for precise quantification of respective cell populations by fluorescence-activated cell sorting (FACS). Using this setup we show wt cells almost completely displace *hsp42*Δ cells, but not *hsp26*Δ cells after 7 days of growth under stress (Fig. 6a). This indicates that the Hsp42-specific aggregase but not the classical Hsp26 ‘holdase' activity provides competitive growth advantage to cells during the applied stress.

In light of the detected stress specific sHsp knockout phenotypes we analysed for the impact of Hsp26 and Hsp42 in facilitating protein disaggregation *in vivo*. We used thermolabile luciferase-mCitrine as disaggregation reporter, as it quantitatively aggregates on heat shock to 45 °C, requires Hsp70/Hsp104 activity for reactivation and allows to measure and correlate several critical parameters: the reactivation kinetics and the disaggregation efficiency.

Luciferase-mCitrine formed aggregates at 45 °C in all cells including *hsp42*Δ, indicating that severe stress conditions outplay dependence on Hsp42 for protein aggregation. Intense luciferase-mCitrine foci formed at 45 °C were, however, less abundant in *hsp42*Δ cells and luciferase-mCitrine staining appeared more diffuse (Supplementary Fig. 11d), indicating that Hsp42 still exerts aggregase activity during severe heat shock. wt and *hsp42*Δ cells showed similar kinetics and yields of luciferase-mCitrine refolding during stress recovery, resulting in 70% reactivation within 30 min (Fig. 6b). In contrast, refolding of aggregated luciferase-mCitrine was delayed two-fold in *hsp26*Δ cells such that maximal luciferase recovery required 60 min (Fig. 6b). Luciferase levels were similar in all tested cells subjected to heat stress, excluding increased proteolysis of luciferase as reason for the refolding delay in *hsp26Δ* cells. The refolding delay instead points to a defect in protein disaggregation (Supplementary Fig. 11a). Indeed, the vast majority (83%) of *hsp26*Δ cells still contained aggregated luciferase-mCitrine, apparent as fluorescent foci, after 30 min of recovery whereas luciferase-mCitrine disaggregation was almost complete in wild type and *hsp42*Δ cells (Fig. 6c; Supplementary Fig. 11b–d). This demonstrates a dominant function of Hsp26 facilitating protein disaggregation.

## Discussion

We used a range of experimental approaches to establish key molecular features of the interactions of Hsp42 and Hsp26 with misfolded proteins and to unravel possible differences between these chaperones in stress-related functions. Since Hsp26 and Hsp42 are the only sHsp members of *S. cerevisiae*, our analysis may serve as model for sHsp function and diversification in a eukaryotic cell.

Both sHsps share the remarkable feature to bind misfolding proteins during early stages of unfolding. Substrates are thereby trapped in minimally misfolded, near-native conformation. Within the oligomeric sHsp–substrate assemblies the trapped substrates are furthermore organized such that individual molecules are well separated from each other. Although being kept in near-native state and prevented from direct aggregation by physical separation, the trapped substrates remain stably associated with the sHsps and do not readily dissociate, as evidenced by the lack of substrate transfer to the GroEL-trap. We suggest these features represent the molecular basis for sHsp activity in protein quality control, likely to be conserved in evolution. Accordingly, sHsps sequester misfolded proteins in a ready-to-refold state facilitating subsequent solubilization and refolding by ATP-dependent chaperones. Our study also reveals that the constitutively active Hsp42, but not Hsp26, acts as aggregase, to promote the active sequestration of misfolded proteins. The aggregase function is an important element in the protective response of cells against proteotoxic stress since in the absence of Hsp42, cells show reduced cellular fitness on recurring heat stress. These findings also demonstrate functional diversification among sHsp family members.

The impact of sHsp binding on substrate conformation was determined by several approaches. Hydrogen exchange (HX) experiments revealed that in the absence of sHsps, peptides isolated from heat-induced amorphous MDH aggregates show globally higher HX than corresponding peptides from native MDH, indicating global unfolding. When complexed with either Hsp26 or Hsp42 heat-denatured MDH is globally protected from HX. These findings agree well with previous work suggesting sHsps interact with destabilized substrate states protecting them from further aggregation^45^,^46^,^47^. An important aspect of our analysis was the detection of heterogeneity in HX of individual MDH peptides. When considering the averaged HX protection profile, the MDH structure appears intermediate between the native and unfolded aggregated states, in agreement with earlier findings for plant sHsps^47^. However, our detailed HX analysis of sHsp–MDH complexes formed at variable sHsp:substrate stoichiometries reveals that MDH exists as hitherto unnoticed mixture of the native and aggregated states. Conformational heterogeneity is largest at substoichiometric MDH:sHsp ratios, conditions that do not allow binding of all MDH molecules to sHsps. At higher sHsp concentrations, the MDH population shifts from high to low exchange states. For all peptides detected, the low-exchanging population is almost identical to the native state population of the same peptide. This native state-like HX pattern does not originate from direct sHsp binding to the respective MDH peptides since it is extremely unlikely that such an interaction confers a native-like protection pattern in all cases. We note that compared with Hsp42, Hsp26 is more efficient in conferring HX protection to MDH, a difference not observed with conventional chaperone assays.

DSS crosslinking identified the C-terminal region of MDH as the major site for sHsp interaction. This region is surface-exposed and located distal to the MDH dimer interface. Peptides corresponding to this region show greatest HX exchange in the aggregated state and become partially protected on sHsp binding. We suggest these MDH sites unfold early on temperature increase and are immediately recognized by sHsps. Fast association with sHsps then protects the remaining part of the bound substrate from further unfolding and keeps central parts in stable, near-native state. This activity also reduces intermolecular hydrophobic interactions of unfolded MDH and keeps sequestered substrate molecules at larger distances from each other as shown by FRET.

Single-molecule measurements on MBP provide further insights into the molecular nature of the effects of Hsp42 on substrate conformations. Hsp42 binding promotes native-like MBP folds and suppresses off-pathway interactions and aggregation between misfolded and aggregated MBP repeats within the 4MBP construct. This distinguishes Hsp42 from other chaperones tested in single-molecule experiments including SecB, which bound to denatured MBP and suppressed aggregation but did not promote native-like folds^35^. Hsp42 activity more closely resembles that of Trigger factor, which suppressed aggregation and promoted folding of MBP^36^. Trigger factor, however, bound a range of structures smaller than one domain, whereas Hsp42 bound the near-native core structure of MBP, and trigger factor increased resistance against forced unfolding, whereas Hsp42 decreased this resistance. Note that mechanical resistance is not the same as thermodynamic stability, and hence cannot be directly compared. For instance the former may be particularly sensitive to intra-molecular contacts at the chain termini. The decreased mechanical resistance conferred by Hsp42 could indicate that the MBP core is close to the unfolding transition state when bound to Hsp42. Hsp42 binding could also induce global structural changes in the MBP core, as seen in allosteric transitions, that decrease its mechanical resistance, or compete with intra-molecular MBP residue contacts that are key to resistance against forced unfolding.

While Hsp26 and Hsp42 share the ability to bind substrates in near-native states, they play distinct roles in cellular proteostasis. We demonstrate that Hsp26, but not Hsp42, is most important for facilitating the refolding of an aggregated model substrate *in vivo*, taking over the classical sHsp function also observed in other species^20^,^27^,^48^. Hsp42 instead exhibits a unique activity by specifically promoting the formation of cytosolic aggregates (CytoQ, Q-bodies) under physiological stress conditions^21^,^22^. Here, we reconstitute the aggregase activity for misfolded proteins *in vitro*, showing that Hsp42 actively sequesters soluble misfolded proteins into larger inclusions. The Hsp42 aggregase activity is already unleashed under mild denaturation conditions, which likely cause partial instead of global MDH unfolding. This would allow substrate sequestration by Hsp42 early during unfolding stress, even before full denaturation has occurred. We surmise that these *in vitro* denaturation conditions mimic physiological stress regimes known to require Hsp42 for formation of microscopically detectable aggregates^21^,^22^.

The aggregase activity requires the large NTE of Hsp42 (ref. 21). Consistently, we observed increased HX protection throughout the NTE in Hsp42–MDH complexes, indicating direct involvement of the NTE in substrate interaction. Which specific feature of the long NTE enables Hsp42 to act as molecular aggregase requires further analysis.

Importantly, we show that the Hsp42 activity provides cellular fitness during stress conditions. This demonstrates not only important physiological functions of Hsp42 but also the cytoprotective role of Hsp42-organized protein aggregation. These aggregates likely counteract the accumulation of soluble, toxic conformers^49^ and reduce the burden for the protein quality control machinery during stress. Evidence for a protective function of sHsp driven protein aggregation during aging has been recently provided for *C. elegans*^2^. Long-lived *daf2* mutant worms accumulate more insoluble proteins compared with age-matched wild type animals, correlating with a strong increase of specific sHsps in the aggregated protein fraction^2^.

**Figure 7:**
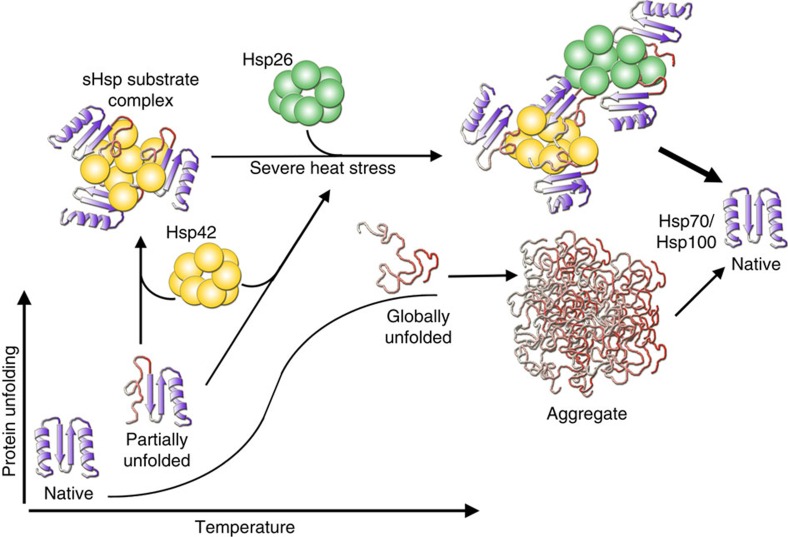
Cellular activities of yeast Hsp26 and Hsp42. During initial protein unfolding Hsp42 sequesters potentially harmful species within sHsp/substrate complexes, thereby triggering the formation of microscopic visible CytoQs *in vivo*. On severe heat stress, temperature-activatable Hsp26 coaggregates, resulting in more efficient dissolution of CytoQs within cells. Both sHsps sequester proteins early during misfolding and aggregation. They preserve a native-like structure of bound substrates and increase the distance between non-native protein molecules, suppressing the formation of tight, large aggregates. These features contribute to facilitated chaperone-mediated disaggregation.

In summary, we show that sHsps preserve native-like substrate conformations and have diversified to also act as aggregase in actively sequestering those states in larger inclusions (Fig. 7). sHsps are therefore not simply chaperones with ‘holdase' function suppressing the formation of large aggregates. Instead, the two yeast sHsps together provide a powerful toolset effectively fighting protein misfolding early on. It is tempting to speculate that the increasing number of sHsps present in metazoan and plants allows for even further diversification of sHsp function in proteostasis networks.

## Methods

### Yeast strains and growth conditions

All *S. cerevisiae* strains used in this study are derived from BY4741 and are listed in Supplementary Table 2. Yeast cultures were cultivated in liquid SC media at 30 °C or at the indicated conditions. The corresponding solid media contained 2% (w/v) agar. To select for resistance to geneticin or nourseothricin, the antibiotic was added to the final concentration of 300 μg ml^−1^ or 100 μg ml^−1^, respectively.

### Proteins

If not stated otherwise, all proteins were produced in derivatives of *E. coli* strain MC4100. Ssa1 and Sse1 were expressed with an N-terminal His_6_-Smt3 tag^50^. The fusion proteins were purified by affinity chromatography using a Ni-IDA sepharose matrix (Macherey-Nagel) according to the manufacturer's protocol. Fused Smt3 was cleaved off by Ulp1 treatment. Ssa1 and Sse1 were separated from Smt3 and Ulp1 by size exclusion using a Superdex200 16/60 column (GE Healthcare). His_6_-tagged MDH, Sis1 and MDH–YFP were purified by affinity chromatography using a Ni-IDA sepharose matrix (Macherey-Nagel) according to the manufacturer's protocol. MDH–YFP was further purified by size exclusion using a Superdex75 16/60 column (GE Healthcare). After Ni chromatography Sis1 containing fractions were pooled, dialysed and contaminants were separated on a ResourceQ column (GE Healthcare). His_6_-GroES and Hsp104-His_6_ were purified by affinity chromatography using a Ni-IDA sepharose, followed by size exclusion using a Superdex200 16/60 column (GE Healthcare). GroEL, and GroEL-Trap were purified by DEAE-Sepharose anion exchange chromatography followed by size exclusion using a Superdex200 16/60 column (GE Healthcare) as also described earlier^51^,^52^.

Hsp26 was overproduced with an N-terminal His_6_-Smt3 tag. Cells were resuspended in buffer A (40 mM HEPES-KOH pH 7.5, 150 mM KCl, 5 % glycerol, 5 mM β-mercaptoethanol), lysed and centrifuged (17,000 r.p.m., 30 min, 4 °C). The pellet was dissolved in buffer A containing 8 M urea by stirring for 2 h at RT. The sample was centrifuged (17,000 r.p.m., 30 min, 4 °C) and the supernatant was incubated with Ni-IDA (Macherey-Nagel) for 1 h at 4 °C. Bound protein was washed with 10 CV of buffer A with 8 M urea, followed by 2 CV of buffer A containing 2 M urea. Protein was eluted with 250 mM imidazole in buffer A with 2 M Urea and Hsp26 containing fractions were pooled. The SUMO-tag was cleaved during dialysis against buffer A at 4 °C over night and the protein was further purified via a Sephacryl S-300 HR 16/60 column (GE Healthcare) equilibrated with buffer A.

C-terminally FLAG-tagged Hsp42 was cloned into pMal-c2E (New England BioLabs) creating a N-terminally fused maltose binding protein (MBP)-tag. The Enterokinase cleavage site was changed to a PreScission cleavage site by site-directed mutagenesis and the vector was transformed into ArcticExpress (Agilent Technologies). Cells were grown at 37 °C to OD_600_ 0.9, 0.5 mM IPTG was added and protein was expressed at 13 °C over night. Cells were resuspended in buffer B (50 mM Tris–HCl, 200 mM NaCl, 2 mM DTT, 10 % glycerol), lysed and centrifuged. The soluble extract was incubated with Amylose resin (New England Biolabs) and the manufacturer's protocol was followed. Hsp42 containing fractions were pooled and the MBP-tag was cleaved at 4 °C over night by PreScission protease (Sigma-Aldrich), followed by size exclusion using a Sephacryl S-300 HR 16/60 column (Amersham) equilibrated in buffer B. Fractions containing Hsp42 were pooled and concentrated by dialysis against buffer B containing 20 % (w/v) PEG 20,000.

MDH used for fluorescent labelling was purchased from Roche and Pyruvate kinase was obtained from Sigma-Aldrich.

### Western blot

GAPDH-antibodies (α-Zwf1) were obtained from Hylest Ltd (cat. SG4) at 1:50,000 dilution. Polyclonal antibodies against MDH, Hsp26, Hsp42 (each at 1:10,000 dilution) and luciferase (at 1:1,000 dilution) were made by Davids Biotechnology using purified proteins. Antibody specificity was documented by use of purified proteins or yeast knockout cells.

### Hydrogen deuterium exchange experiments

Native His_6_-MDH (2 μM, 400 μl), thermally aggregated His_6_-MDH or sHsp/His_6_-MDH complexes (both formed for 30 min at 47 °C) in buffer C (50 mM HEPES pH 7.6, 50 mM KCl, 5 mM MgCl_2_, 2 mM DTT) were incubated with 50 μl MagneHis Ni-Particles (Promega) for 15 min at room temperature. His_6_-MDH (aggregated or sHsp-bound) was isolated by placing the reaction in a magnetic reack. The supernatant was subsequently removed and the beads were washed once with buffer C. D_2_O-based buffer C was added to initiate amide proton-deuteron exchange. After 30 s the exchange reaction was quenched by adding ice-cold low pH quench buffer (500 mM K-phosphate buffer, pH 2.2) containing pepsin (25 μg ml^−1^, Roche). Protein was digested from the Ni-Particles for 1 min on ice. Quenched, digested samples were injected into the HPLC setup, with online peptic digest, and analysed on an electrospray ionization quadrupole time-of-flight mass spectrometer (QSTAR Pulsar, Applied Biosystems) as described in ref. 53. Analysis of deuteron incorporation into peptides was performed by using AnalystQS software (Applied Biosystems/MDS SCIEX, Germany). The assignment of the isotope peaks and the selection of the peptides presented were done manually.

### Thermal shift assay

Thermal MDH unfolding was monitored by measuring the increase in the fluorescence of the fluorophore SYPRO orange (Sigma-Aldrich) on binding to hydrophobic protein patches. Overall, 20 μl of MDH (2 μM) in buffer C were incubated in a 384-well microplate and fluorescence was monitored by a real-time PCR device (LightCycler 480 II, Roche). The sample was heated at 2 °C per min, from 20 to 95 °C.

### Aggregation assays

#### Light scattering

For light scattering measurements 0.5 μM MDH in buffer C was denatured at 47 or 41 °C in the absence or presence of various sHsp concentrations. Turbidity was measured at an excitation and emission wavelength of 550 nm (47 °C) or 600 nm (41 °C), respectively (Perkin-Elmer luminescence spectrometer LS50B).

#### Centrifugation/supernatant-pellet assay

To test for the chaperone function of sHsps MDH (2 μM) in buffer C was denatured for 30 min at 47 °C in the presence or absence of sHsps followed by 30 min centrifugation (14,000 r.p.m., 4 °C). Supernatants and pellets were analysed by SDS–PAGE followed by Coomassie staining. To test for the aggregase function of sHsps MDH (0.5 μM) in buffer C was denatured for 60 min at 41 °C in the presence or absence of sHsps followed by 30 min centrifugation (14,000 r.p.m., 4 °C). Supernatants and pellets were analysed by SDS–PAGE and western blot analysis.

#### Glycerol gradient centrifugation

MDH (0.5 μM) in 100 μl buffer C was denatured for 60 min at 41 °C in the presence or absence of sHsps. The samples were loaded onto a 10–50% glycerol gradient prepared in buffer C+1 mM BSA. The tubes were ultracentrifuged at 40,000 r.p.m. for 1 h at 4 °C. Overall, 600 μl fractions were collected and analysed by SDS–PAGE followed by western blot analysis.

### Disaggregation and refolding of thermally aggregated MDH

MDH (0.5 or 2 μM) was denatured in buffer C for 30 min at 47 °C or for 60 min at 41 °C, respectively. Protein disaggregation and refolding were started by diluting aggregated proteins or sHsp/protein complexes and chaperones 1:1 (2 μM Ssa1, 1 μM Sis1, 0.1 μM Sse1, 1 μM Hsp104, 1 μM GroEL, 1 μM GroES) in buffer C containing 0.1 mg ml^−1^ BSA at 30 °C. The reactivation was monitored as published previously^16^ using an Amersham Biosciences Novaspec Plus spectrophotometer. The same assay was used to follow the inactivation of MDH (0.5 μM) in the presence or absence of sHsps on incubation at 47 °C or 41 °C, respectively.

### ^3^H labelling of His_6_-MDH and size exclusion chromatography

Radioactive labelling was performed by incubating His_6_-MDH with N-succinimidyl [2,3-^3^H] propionate (Amersham; 40 Ci per mmol) for 3 h at room temperature in buffer C. Free unreacted N-succinimidyl [2,3-^3^H] propionate was removed by dialysis against buffer C. Overall, 1 μM ^3^H-MDH and 5 μM of sHsps were heat treated for 30 min at 47 °C. The formed sHsp/^3^H-MDH complexes were incubated for 10 min at 30 °C with 2 mM ATP in the presence or absence of 14 μM GroEL-trap. As a control ^3^H-MDH was aggregated in the presence of 2 mM ATP and 14 μM GroEL-trap (30 min at 47 °C). All samples were separated at room temperature by Superose 6 10/300 GL (GE Healthcare) size exclusion chromatography in buffer C containing 5% (v/v) glycerol. Collected fractions were quantified by scintillation counting. sHsp/MDH complex formation was also monitored by SDS–PAGE and Coomassie staining. Here, Hsp26 (12.5 μM) or Hsp42 (20 μM) was incubated for 30 min at 47 °C in presence or absence of MDH (10 μM). sHsps and sHsp/MDH complexes were separated by S200 HR10–30 (Amersham Biosciences) size exclusion chromatography in buffer C at 4 °C.

### FRET during thermal aggregation of MDH

MDH (Roche) was labelled with 7-diethylcoumarin-3-carboxylic acid succinimidyl ester (Molecular Probes) according to the manufacturer's protocol. The labelled MDH and a C-terminally YFP-tagged MDH variant (each 0.25 μM) were mixed in preheated buffer C and the FRET (Förster energy resonance transfer) signal was recorded at 527 nm in a Perkin-Elmer (Germany) LS50B spectrofluorimeter at 47 °C.

### Crosslinking mass spectrometry

*DSS crosslinking*. Native His_6_-MDH (5 μM, 200 μl), thermally aggregated His_6_-MDH or sHsp/His_6_-MDH complexes (both formed for 30 min at 47 °C) in buffer C were mixed with 2.7 μl DSS stock solution (1.25 mM each of DSS-d0 and 1.25 mM DSS-d12 in DMF) (Creative Molecules, Canada). Samples were incubated for 30 min at 30 °C in an Eppendorf Thermomixer mixing at 300 r.p.m. Remaining cross-linker was quenched by adding aqueous NH_4_HCO_3_ to a final concentration of 90 mM and incubation for 10 min at 35 °C and 600 r.p.m. RapiGest SF Surfactant (Waters), urea and NH_4_HCO_3_ were added to final concentrations of 1 μg μl^−1^, 8 and 300 mM, respectively. Samples were sonicated for 1 min, DTT was added (10 mM final concentration) followed by 30 min incubation at 37 °C and 600 r.p.m. Aqueous iodacetamide (GE Healthcare) solution was added to a final concentration of 15 mM. After incubation for 30 min at RT in the dark, DTT was added (final concentration 10 mM) for 5 min at RT. Subsequently, lysyl endopeptidase (mass spectrometry grade; Wako Chemicals) was added at an enzyme-to-substrate ratio of 1:100, followed by incubation for 6 h at 37 °C and 600 r.p.m. The solution was adjusted to 2 M urea and trypsin (ThermoScientific) was added at a 1:50 enzyme-to-substrate-ratio. After incubation over night at 37 °C and 600 r.p.m., samples were acidified to 2% formic acid and purified by solid-phase extraction using 50 mg Sep-Pak tC18 cartridges (Waters) according to the manufacturer's protocol. The eluate was evaporated to dryness in a vacuum centrifuge.

*Fractionation of crosslinked peptides*. Samples were solved in 50 μl of SEC mobile phase (water/acetonitrile/trifluoroacetic acid, 70:30:0.1) and size exclusion chromatography was performed as previously described^54^.

*Liquid chromatography-tandem mass spectrometry*. Peptides were reconstituted in 0.1% TFA and analysed by an Orbitrap Elite mass spectrometer (Thermo) coupled to an UltiMate 3000 RSLCnano System (Thermo). After trapping, samples were loaded on a 75 μm × 250 mm Acclaim PepMap (Thermo) column with buffer A (0.1% formic acid (FA), 1% acetonitrile (ACN), 98.9% H_2_O) and eluted with buffer B (0.1% FA, 10% H_2_O, 89.9% ACN). Peptide separation was achieved with a 300 nl min^−1^ flow rate using the following gradient: 0–3 min: 4% B, 3–90 min: 4–45% B, 90–95 min: 45–95% B, 95–101 min 95% B. The mass spectrometer was operated in data dependent mode with the top 20 most intense ions (resolution: 60,000) selected for fragmentation in the range of *m*/*z* 350–1,600 by collision-induced dissociation at 40%. Singly and doubly charged peptides as well as unassigned were excluded and dynamic exclusion duration was set to 60 s, list size of 500 and a mass window of 20 p.p.m.

*Data analysis*. Resulting Thermo Xcalibur raw files were converted to mzXML format using MSConvert (ProteoWizard version 3.0). Crosslinked peptides were analysed using xQuest and xProphet as previously described^54^. In brief, masses of 12.07531 Da difference for DSS-d0 and DSS-d12 were paired requiring a charge state of 3–7 within a 1 min retention time window of triggering. Spectra were searched against a database containing the UniProt entries of the target proteins as well as the reverse sequence. For xQuest the following search parameters were used: Two maximal missed cleavages, peptide length: 4–50 amino acids, fixed modifications: carbamidomethylated Cys, variable modification: oxidized Met, number of variable modification: 1, mass shift of the light cross-linker: 138.0680796, mass shift of mono-links: 156.0786442 and 155.0964278 Da, MS1 tolerance: 10 p.p.m., MS2 tolerance: 0.2 Da for common and 0.3 Da for crosslink ions. For filtering the search results a target/decoy false discovery rate of 5% was estimated using xProphet as described^55^; the following filtering criteria were used: MS1 mass tolerance window: −4 to 7 p.p.m., ld score >20, min delta score of 0.95.

### Single-molecule optical tweezers assay

For optical tweezers experiments, the protein molecules and DNA handles were immobilized on the bead surfaces using antigens-antibodies connections, as reported previously^25^. Briefly, single MBP and 4MBP molecules were tagged with four c-myc tags at C terminus and were attached to Anti-c-myc-coated beads. The 2,553 bps DNA handles, generated by PCR using a digilated and a biotinylated primer, were connected to AntiDig-coated beads. The immobilized proteins N terminal were linked to the DNA handles via a biotin–neutravidin–biotin connection. In the 4MBP single-molecule experiments, we classify observed unfolding events according to the unfolding force *F* and the change in contour length **Δ***L*, which is determined by fitting the force-extension data to the worm-like chain model for non-interacting polymers (Supplementary Table 3).

### Competition assays

*S. cerevisiae* wild type cells, expressing BFP, and respective mutant cells, expressing GFP, were grown to mid-log phase. Equal amounts of both cultures were mixed and diluted to OD_600_ 0.05. The mixed culture was split into two tubes (halves) and both were grown for seven days with daily dilutions to OD_600_ 0.05. One culture was constantly grown at 30 °C, the other culture was subjected to temperature cycles switching between 1 h at 25 °C and 1 h at 43 °C. Each day the proportion of wild type and mutant cells was measured by monitoring the fraction of GFP- and BFP-positive cells by FACS analysis (BD FACSCanto II, BD Biosciences).

### *In vivo* luciferase refolding

*S. cerevisiae* cells expressing thermolabile luciferase (yEmCitrine-luciferase) in the respective genetic background were grown at 30 °C to mid-log phase. For a pre-shock, cells were shifted to 37 °C for 45 min and subsequently they were heat-shocked at 45 °C for 20 min. *De novo* synthesis of luciferase was inhibited by addition of 10 μg ml^−1^ cycloheximide before heat shock. Reactivation of luciferase was allowed on a shift to 30 °C and was monitored using a Lumat LB 9507 luminometer (Berthold Technologies). An appropriate amount of cell culture was transferred to Ø12 mm polystyrene vials and mixed with 100 μl of 250 μM luciferin. After a lack time of 5 s, light emission was recorded for 10 s. The luciferase activity before heat shock was set as 100% and the measured relative light units were normalized according to the OD_600_ of the respective culture.

### Fluorescence microscopy

To perform live cell imaging, over-night yeast cultures were diluted into fresh medium to an OD_600_=0.05–0.1 and further cultivated at 30 °C to the exponential growth phase of OD_600_=0.5. The cultures were shifted to pre-warmed water bath (37 °C) for 45 min. Next, cyclohexmide was added to a final concentration of 10 μg ml^−1^ to inhibit protein synthesis. Cultures were subsequently shifted to 45 °C for 20 min. Afterwards, cells were allowed to recover at 30 °C. Samples were taken at indicated time points and immediately inspected on Olympus IX81 microscope equipped with a Plan-Apochromat 100 × /1.45 NA oil objective and an EMCCD Hamamatsu camera.

### Data availability

The authors declare that the data supporting the findings of this study are either available within the article (and its Supplementary Information File) or available from the corresponding authors on request.

## Additional information

**How to cite this article:** Ungelenk, S. *et al*. Small heat shock proteins sequester misfolding proteins in near-native conformation for cellular protection and efficient refolding. *Nat. Commun.* **7,** 13673 doi: 10.1038/ncomms13673 (2016).

**Publisher's note**: Springer Nature remains neutral with regard to jurisdictional claims in published maps and institutional affiliations.

## Supplementary Material

Supplementary Information Supplementary Figures 1-13 and Supplementary Tables 1-3.
