# Supplementary material for: Small heat shock proteins sequester misfolding proteins in near-native conformation for cellular protection and efficient refolding
Source: Nat Commun. 2016 Nov 30;7:13673. doi: 10.1038/ncomms13673 (PMC5141385; doi:10.1038/ncomms13673)
Supplement: Supplementary Information — Supplementary Figures 1-13 and Supplementary Tables 1-3. [file ncomms13673-s1.pdf]

# Supplementary Figure 1

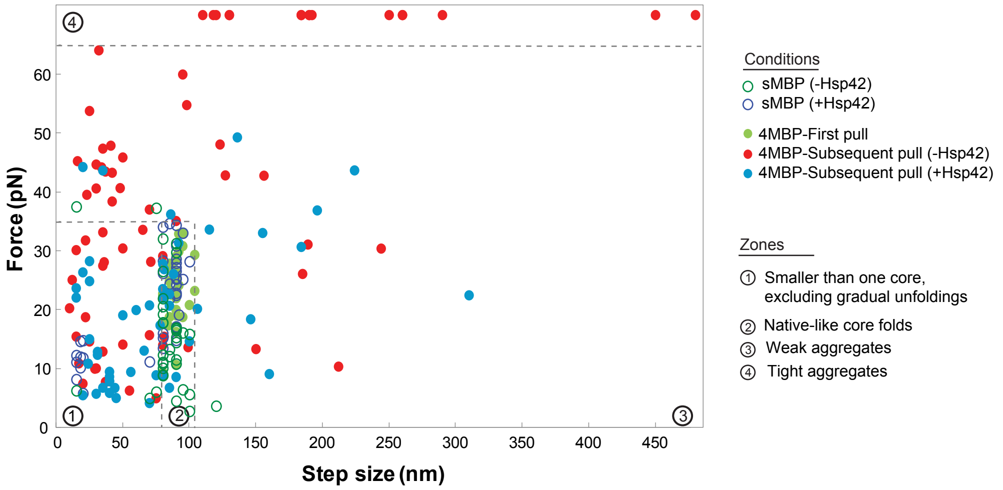


## Supplementary Figure 1

Scatter plot of unfolding forces and step sizes observed in the presence and absence of Hsp42, for sMBP and 4MBP constructs. Note that the gradual unfolding events (of 4MBP construct), are not represented in this figure.

## Supplementary Figure 2

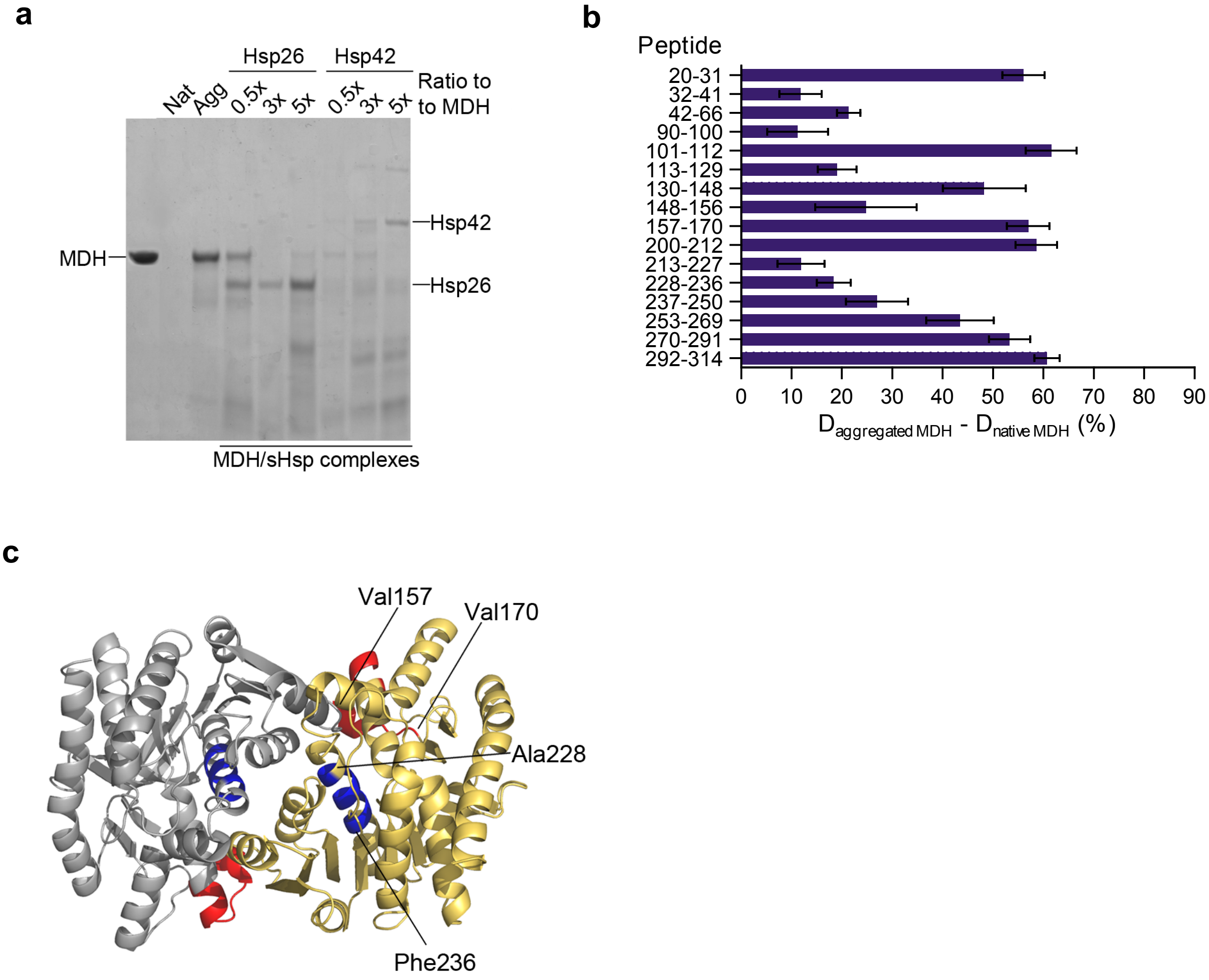


### Supplementary Figure 2

Analysis of MDH aggregate structure by HX. (**a**) Pepsin degrades aggregated MDH. Pepsin digest of resin-bound $His_6$-MDH (native (Nat), aggregated (Agg) or complexed with Hsp26 or Hsp42) was analyzed by SDS-PAGE and Coomassie staining. (**b**) Difference in deuteron incorporation after 30 s between aggregated and native MDH at the peptic peptide level. (**c**) Peptides Ala228-Phe236 (blue) and Val157-Val170 (red) are located at the dimer interface in native MDH. Error bars denote standard deviations for each point based on three repetitions.

## Supplementary Figure 3

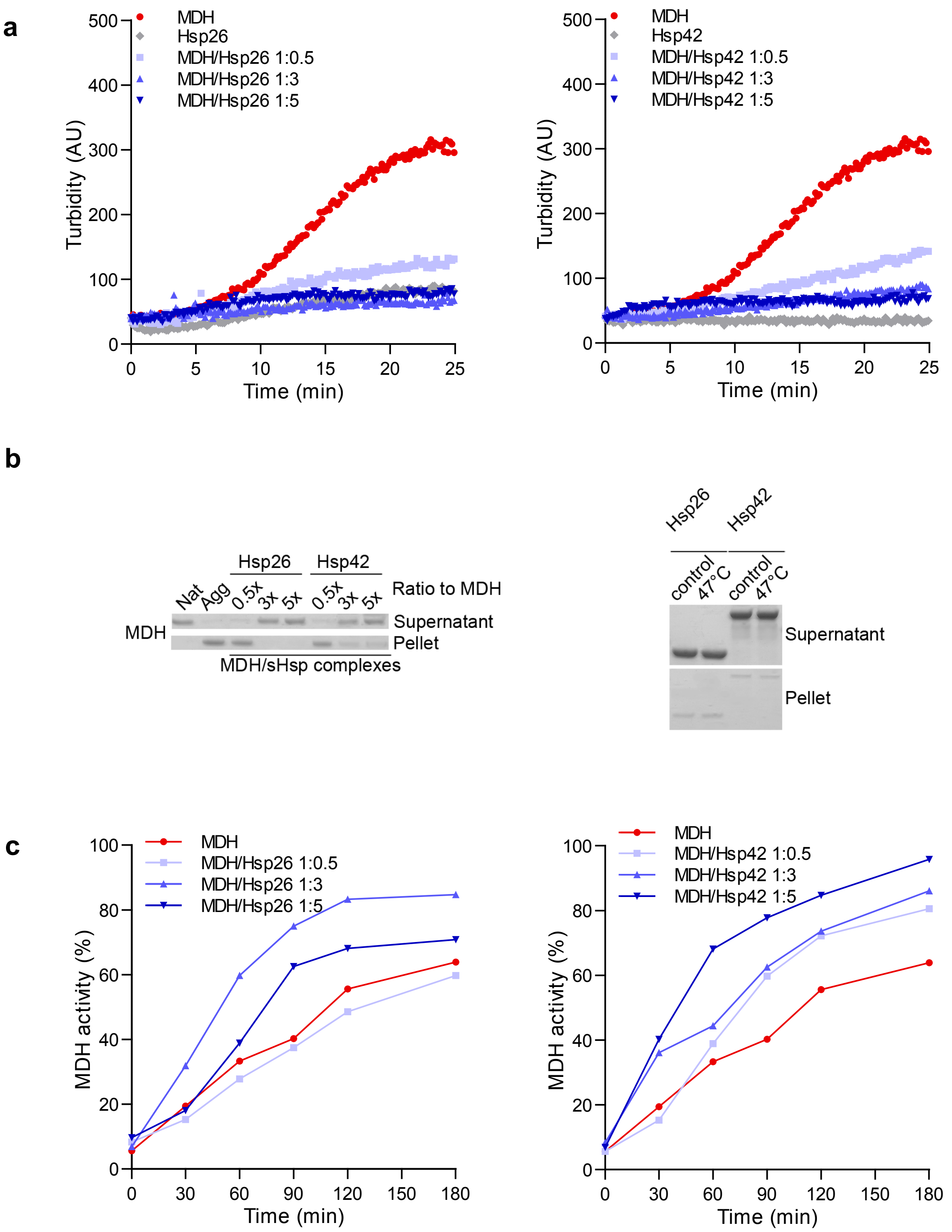

**Supplementary Figure 3**

Hsp26 and Hsp42 prevent the formation of turbid, insoluble MDH aggregates and facilitate chaperone-mediated MDH refolding. (**a)** MDH (0.5 μM) was denatured for 30 min at 47°C in the absence or presence of sHsps at various ratios (0.25 – 2.5 μM). As control 2.5 μM sHsps was heated alone. The formation of turbid MDH aggregates was followed at 550 nm. (**b**) Samples were prepared as described above and centrifuged (30 min, 13 000 rpm, 4°C). Equal amounts of supernatants and pellets were analyzed by SDS-PAGE and Coomassie-staining. (**c**) MDH refolding from aggregated or sHsp-complexed states was initiated at 30°C by addition of the *S. saccharomyces* bichaperone system (2 μM Ssa1, 1 μM Sis1, 0.1 μM Sse1, 1 μM Hsp104) and 1 μM GroEL/GroES. MDH activities were determined at the indicated time points. The enzymatic activity of native MDH was set at 100 %.

## Supplementary Figure 4

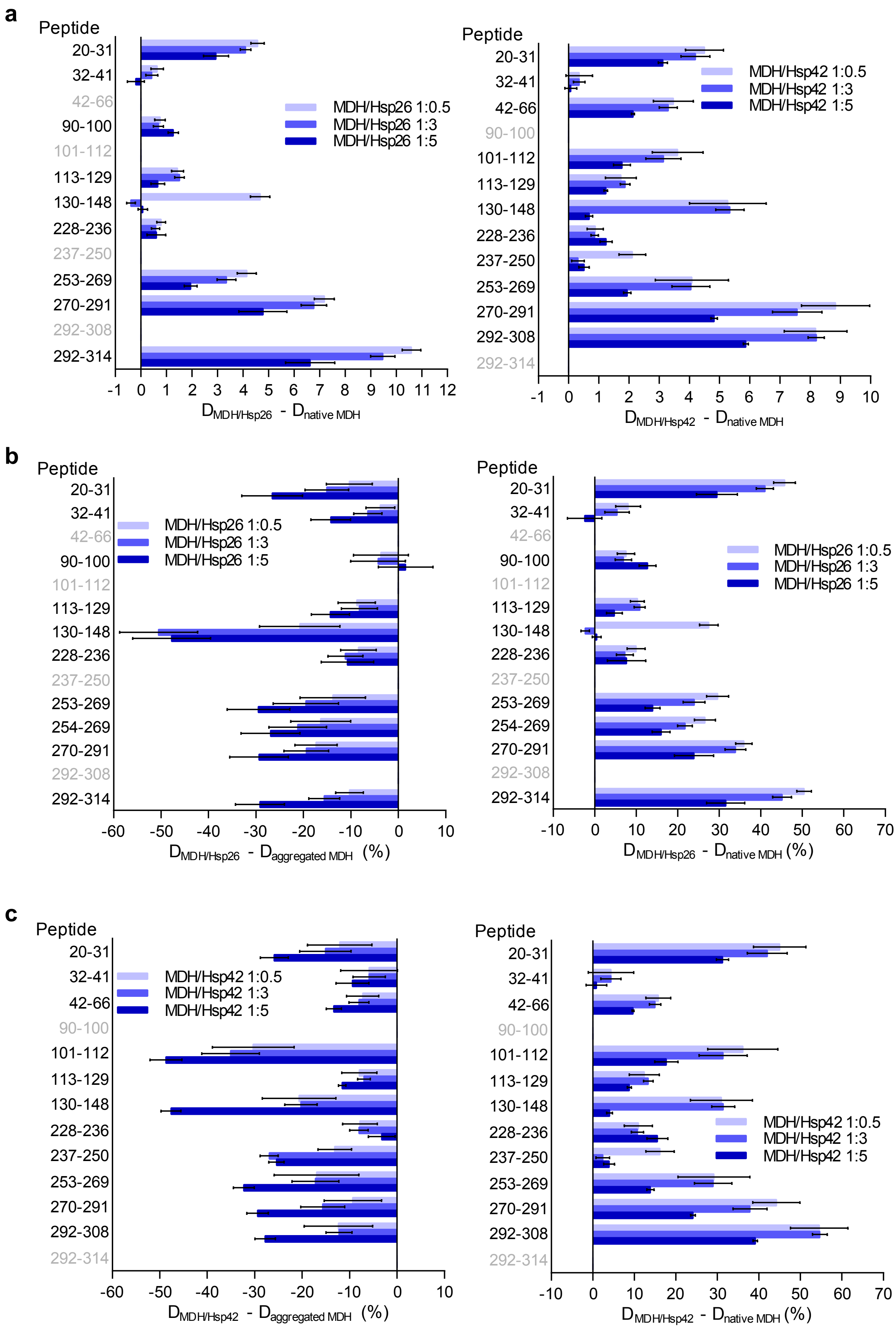

**Supplementary Figure 4**

Hsp26 and Hsp42 protect unfolded regions of aggregated MDH from HX. (**a-c**) Difference in deuteron incorporation into peptic fragments of MDH in heat-induced MDH/Hsp26 or MDH/Hsp42 complexes and aggregated or native MDH. Peptides in gray could not be identified. Error bars denote standard deviations for each point based on three repetitions.

## Supplementary Figure 5

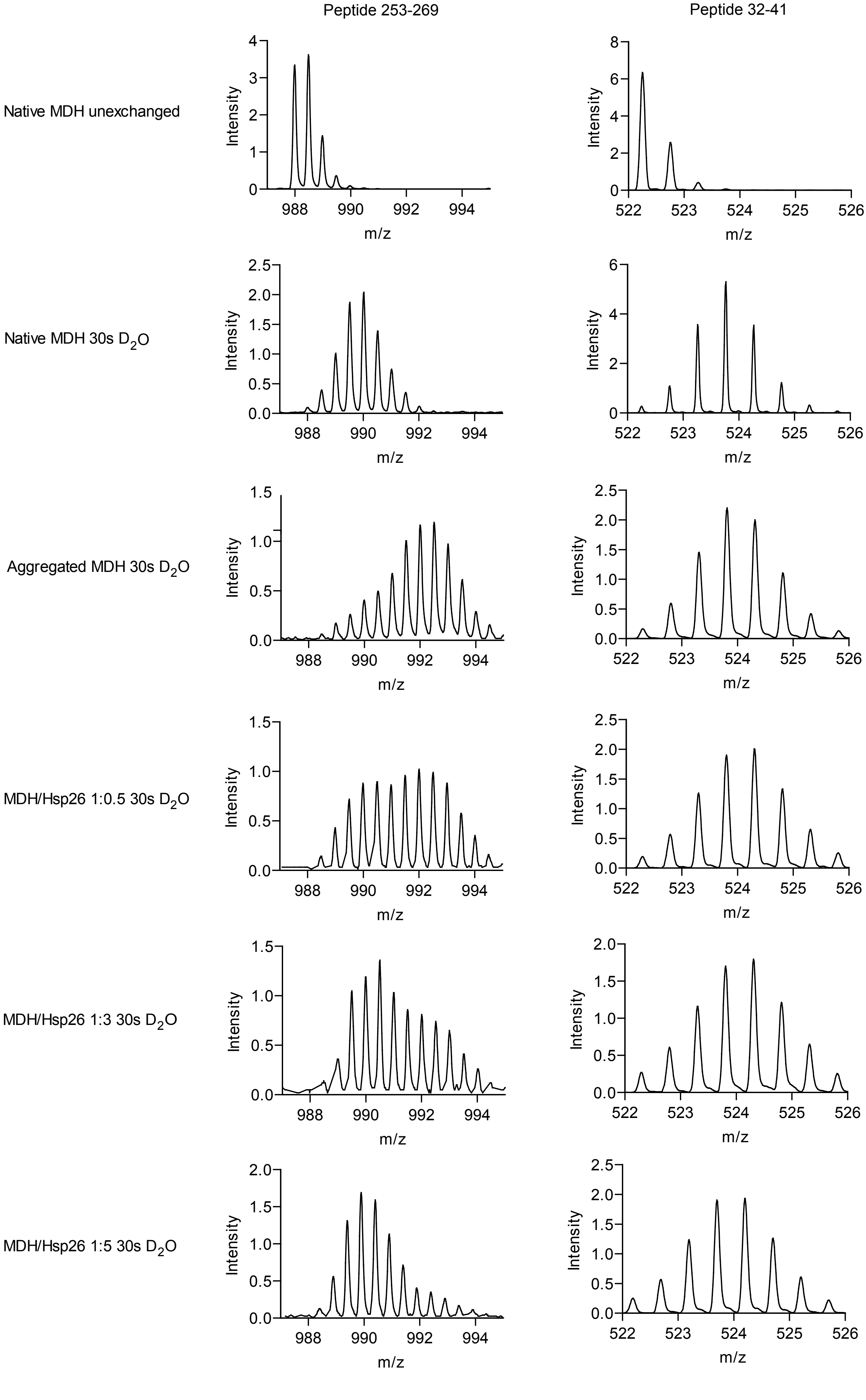

**Supplementary Figure 5**

sHsps stabilize segments of bound MDH in a native-like state. Mass spectra of MDH peptides in the native, aggregated or Hsp26-complexed states after 30 s $D_2O$ incubation at 30°C. The left panels show a representative peptide displaying a bimodal distribution after HX when complexed with sHsps, whereas the peptide shown in the right panels exists as a single population.

## Supplementary Figure 6

**a**

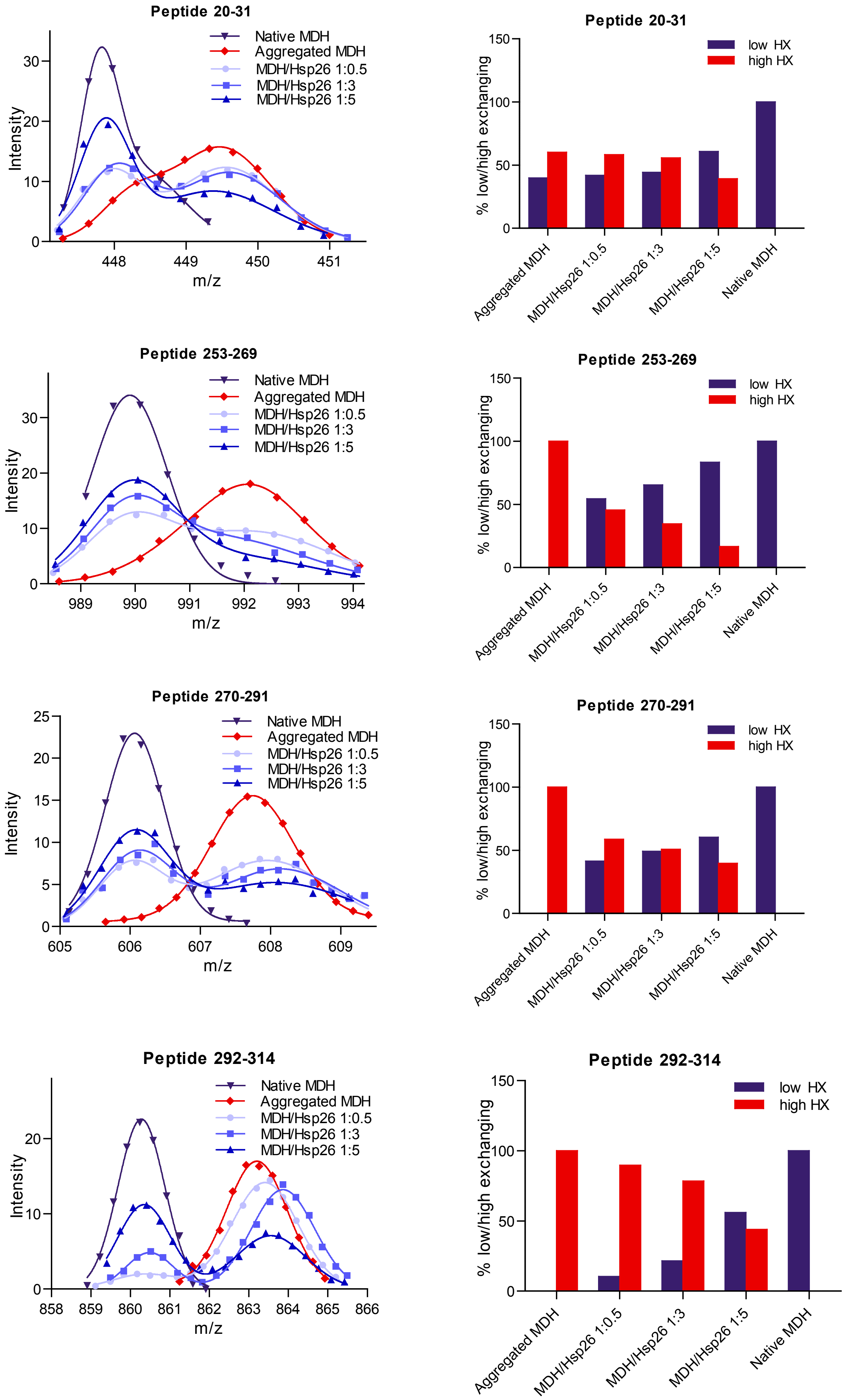

## Supplementary Figure 6

**b**

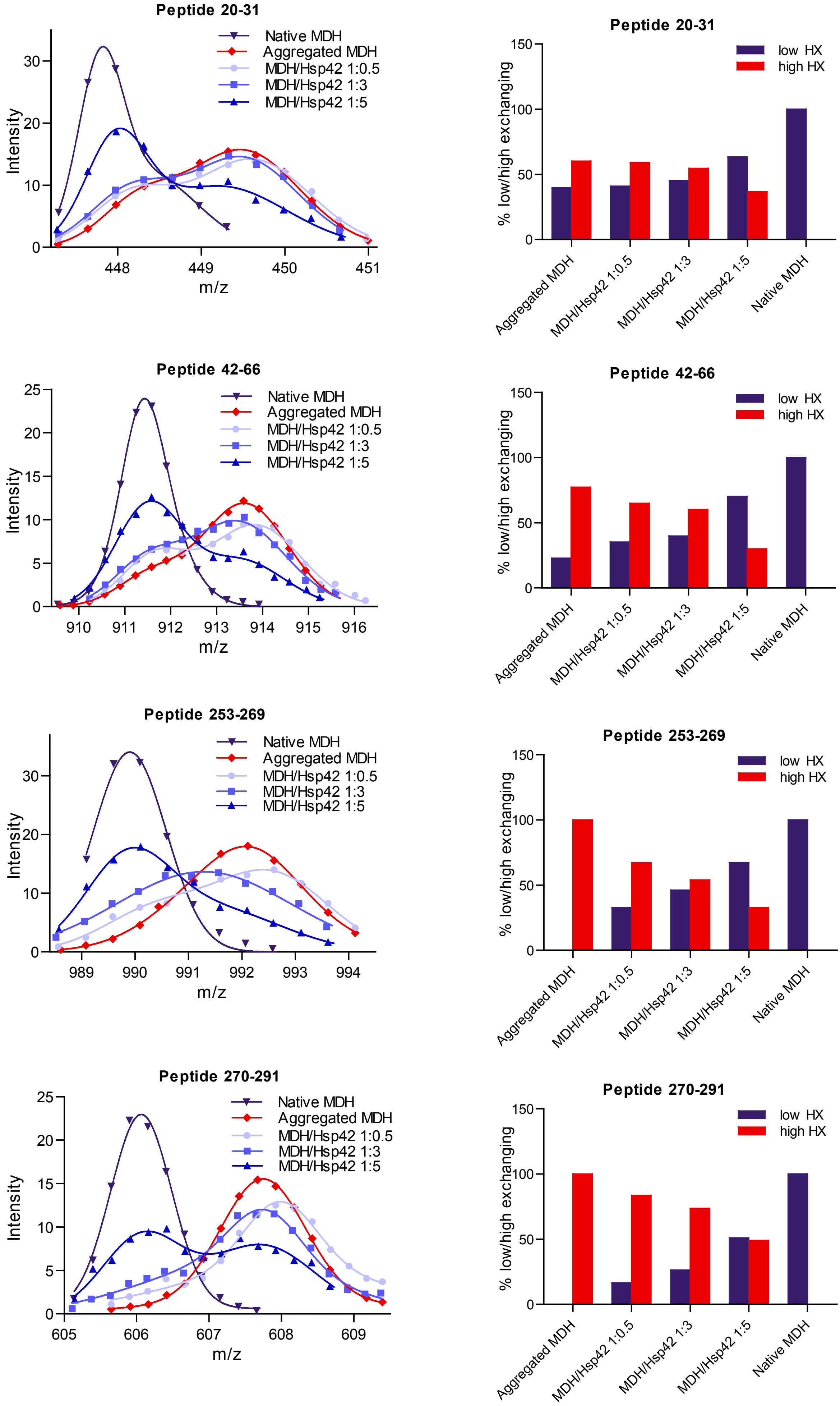

## Supplementary Figure 6

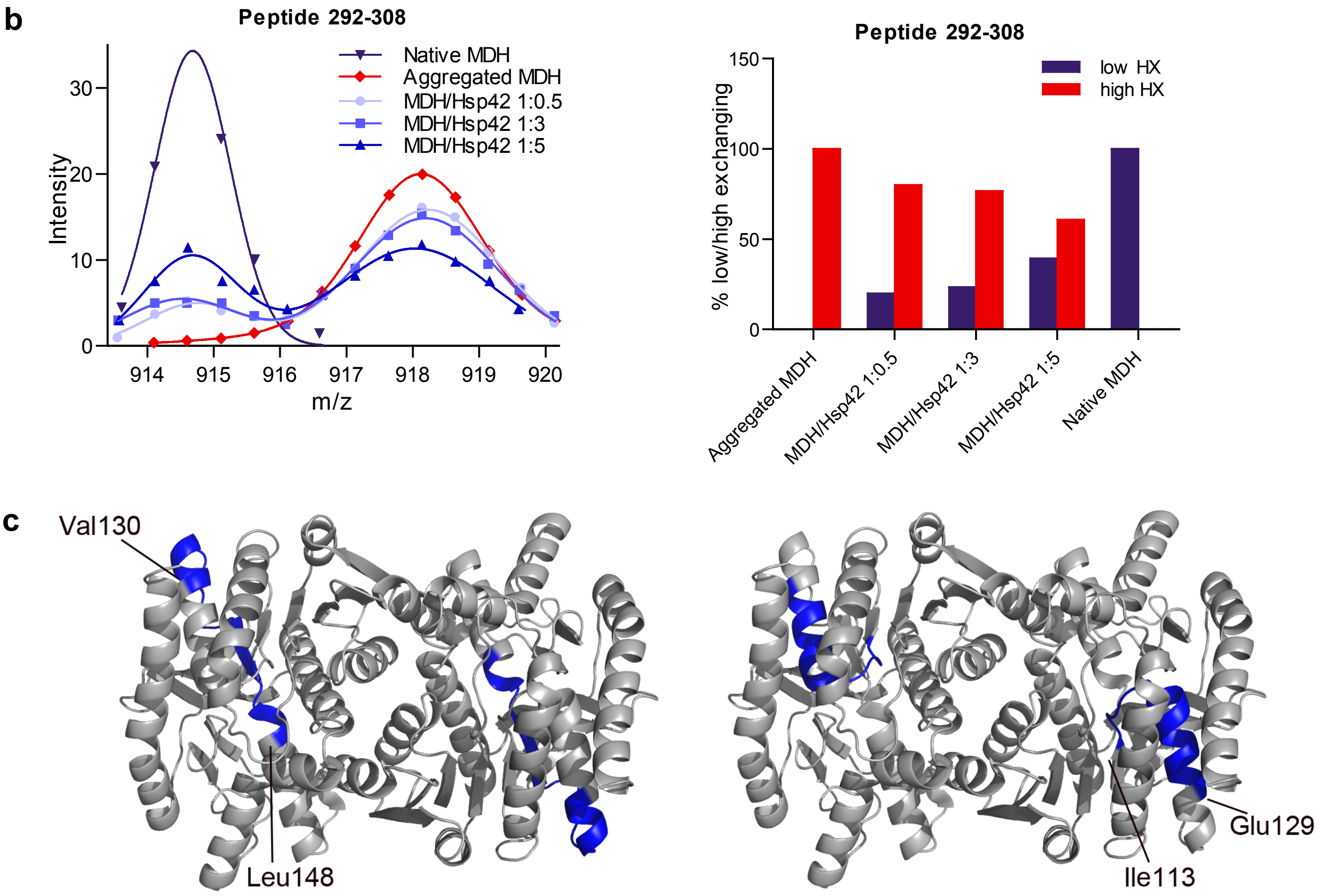


**Supplementary Figure 6**

sHsps stabilize segments of bound MDH in a native-like state. Bimodal distribution of isotope peaks of indicated MDH peptides derived from MDH/Hsp26 (**a**) and MDH/Hsp42 (**b**) complexes. Left panels: Intensity versus m/z diagrams after 30 s HX at 30°C. Right panels: Fractions of native-like and aggregate-like populations calculated for respective peptides. (**c**) Localization of MDH peptides (blue) showing almost exclusively native-like HX patterns when bound to sHsps in the MDH dimer structure.

**Supplementary Figure 7**

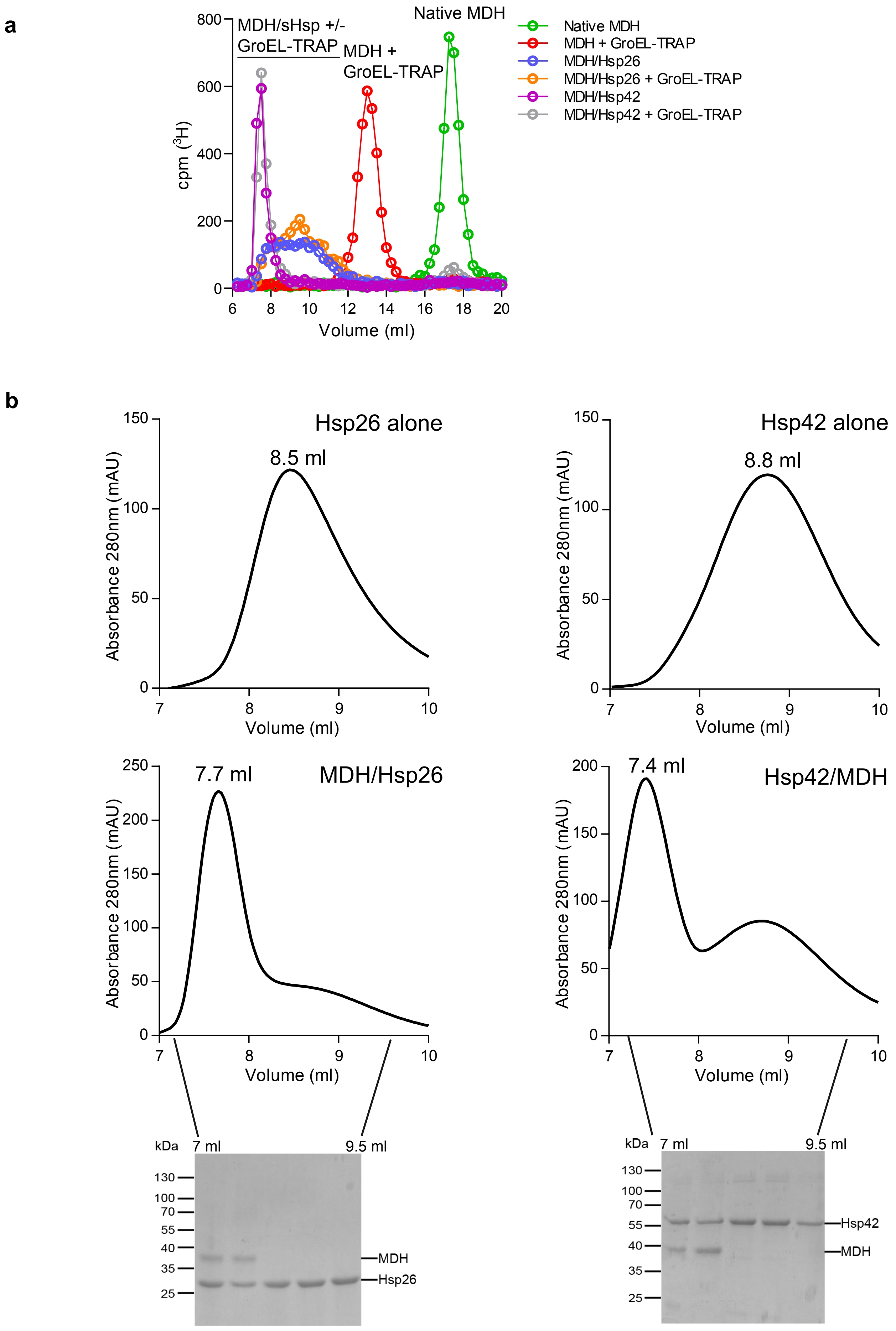

**Supplementary Figure 7**

MDH does not spontaneously dissociate from MDH/Hsp26 and MDH/Hsp42 complexes. (**a**) 1 µM $^3$H-MDH and 5 µM sHsps were heat treated for 30 min at 47°C. The formed sHsp/protein complexes were incubated for 10 min at 30°C with 2 mM ATP in the presence or absence of 14 µM GroEL-D87K (GroEL-trap). As control $^3$H-MDH was denatured in presence of 2 mM ATP and 14 µM GroEL trap (30 min at 47°C). All samples were separated by size exclusion chromatography and collected fractions were quantified by scintillation counting (open circles). (**b**) Hsp26 (12.5 µM) or Hsp42 (10 µM) was incubated for 30 min at 47°C in presence or absence of MDH (5 µM). sHsps and sHsp/MDH complexes were separated by size exlusion chromatography (S200 HR10–30). Sec fractions were analyzed by SDS-PAGE followed by Coomassie staining.

## Supplementary Figure 8

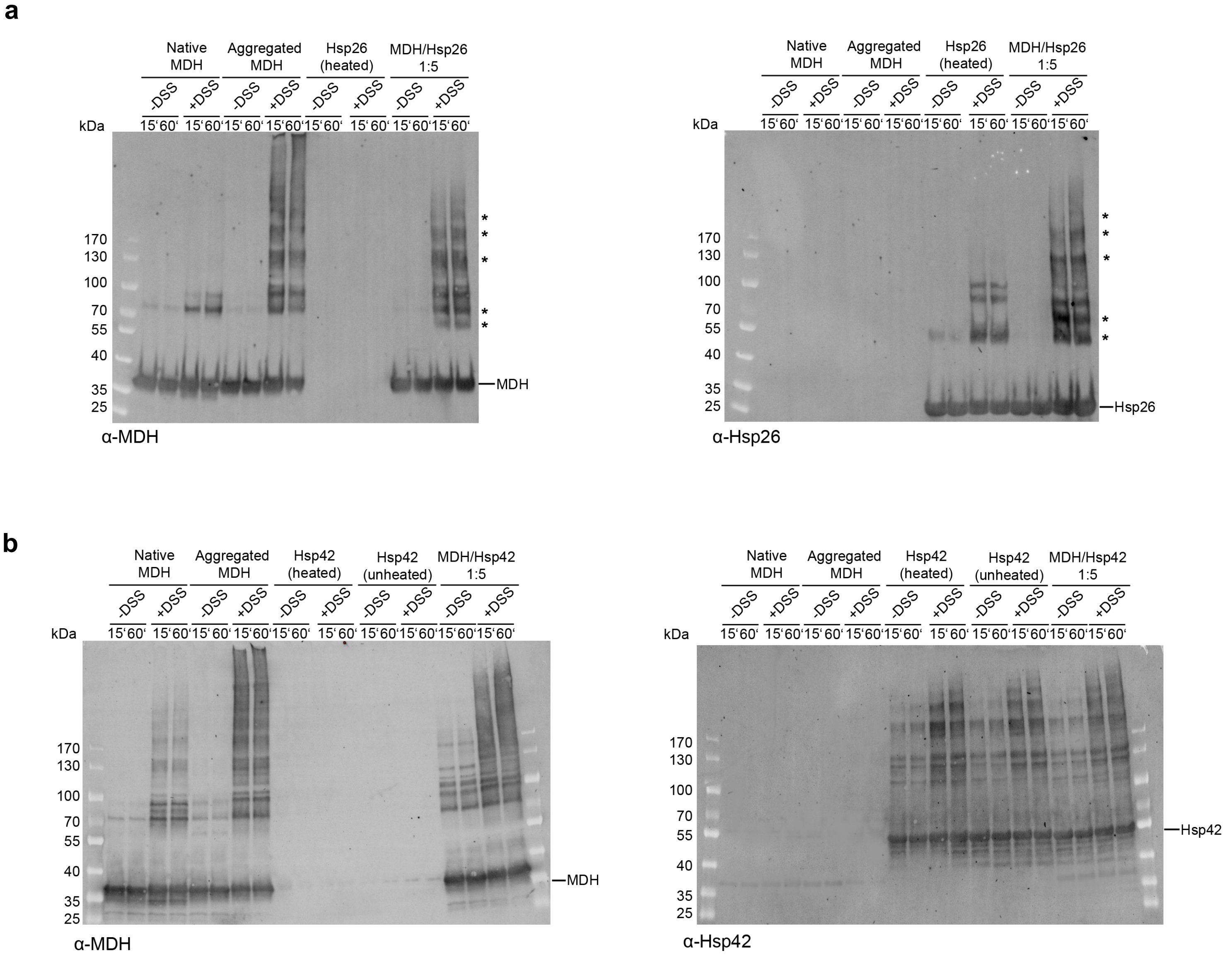


**Supplementary Figure 8**

DSS-crosslinking of native MDH, MDH aggregates and MDH/sHsp complexes. MDH (native or aggregated) and MDH/sHsp 1:5 complexes (**a**) Hsp26 and (**b**) Hsp42 were incubated with or without DSS at 30°C for 15 and 60 min. Crosslink products were analyzed by western blotting using MDH, Hsp26 and Hsp42 specific antibodies. Crosslinks between MDH and Hsp26 are indicated by asterisks.

## Supplementary Figure 9

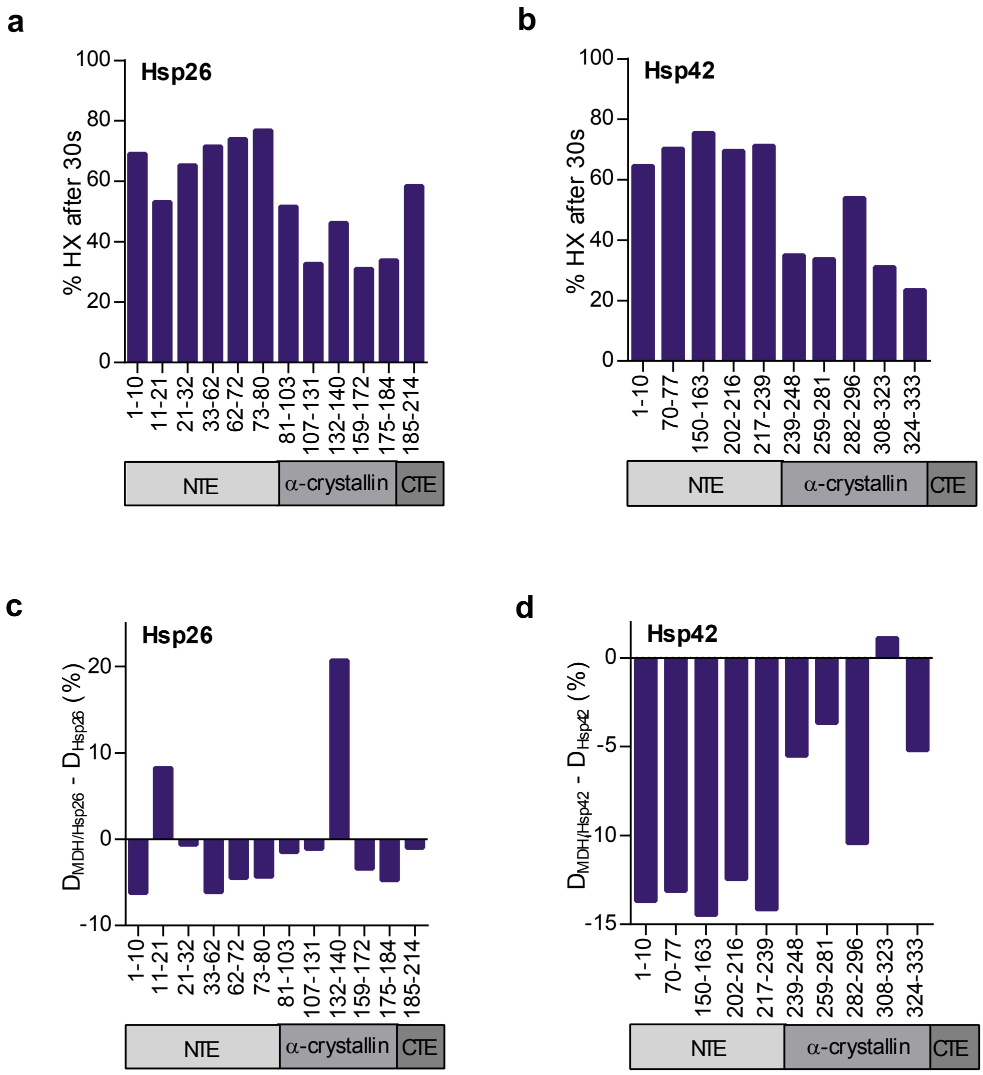


**Supplementary Figure 9**

Analysis of free and substrate-complexed sHsps states by HX. Deuteron incorporation into sHsps after 30 s incubation in $D_2O$ at 30°C. HX pattern of free Hsp26 (**a**) or Hsp42 (**b**) and difference in deuteron incorporation between MDH-complexed and free Hsp26 (**c**) or Hsp42 (**d**). Hsp26-MDH and Hsp42-MDH complexes were generated by incubating MDH at 47°C in presence of 5-fold excess of sHsps and separated from free sHsps by size exclusion chromatography. All data were corrected for deuteron losses due to back-exchange using a 100% deuterated control. NTE: N-terminal extension, CTE: C-terminal extension.

## Supplementary Figure 10

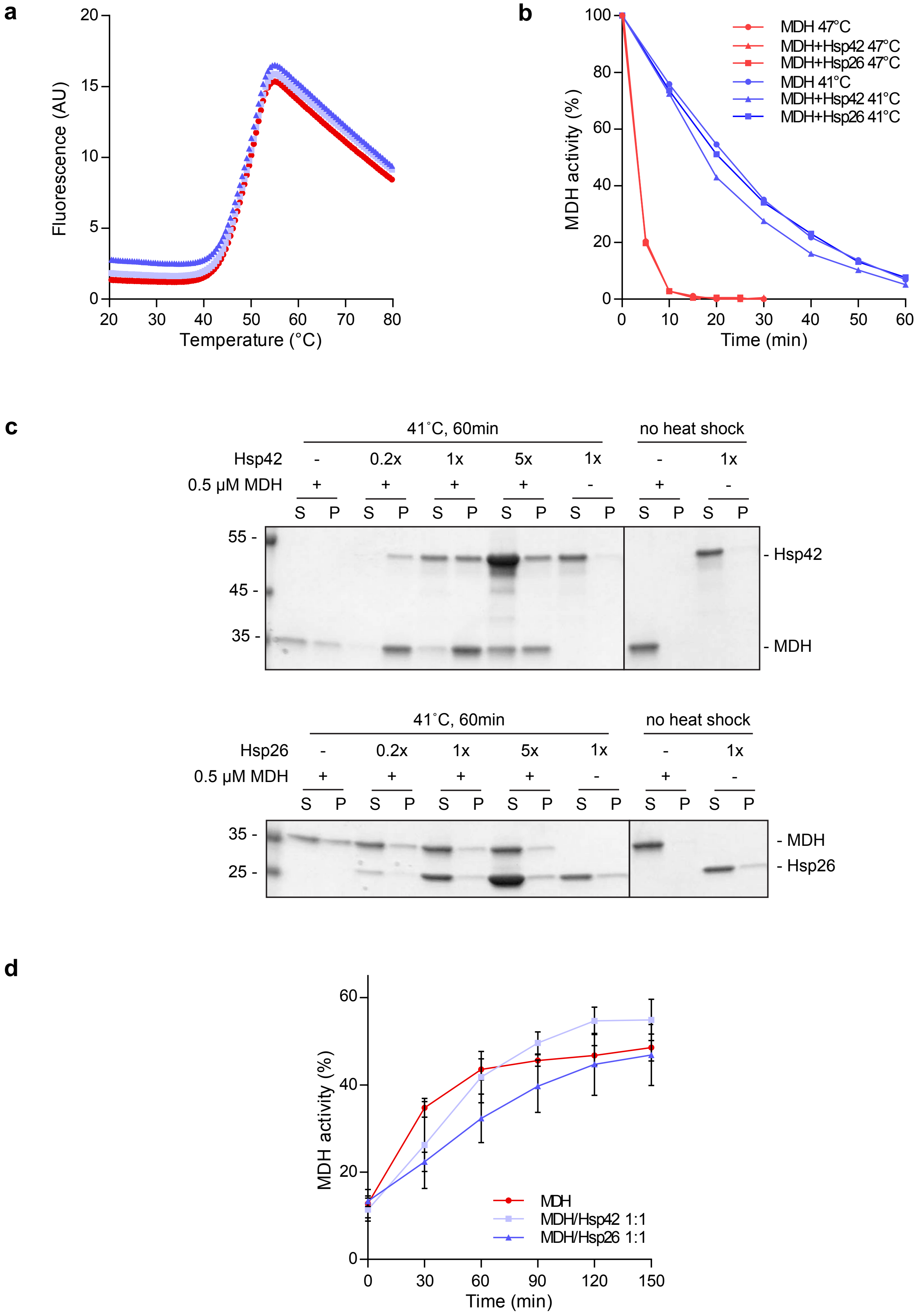

**Supplementary Figure 10**

Hsp42-promoted aggregation during initial protein unfolding does not affect protein recovery. (**a**) Thermal denaturation curve of MDH. The binding of SYPRO orange to hydrophobic patches leads to enhanced fluorescence upon protein denaturation at increasing temperature. Fluorescence intensities were measured in a RT-PCR-device in the range of 20-95°C. The melting point of MDH was calculated to 50.9°C. (**b**) MDH (0.5 µM) was incubated at 41°C in the presence and absence of sHsps and the loss of MDH activity was measured. (**c**) MDH (0.5 µM) was incubated for 1 h at 41°C in the presence or absence of sHsps and centrifuged (30 min, 13 000 rpm, 4°C). Equal amounts of supernatants and pellets were analyzed by SDS-PAGE and Coomassie-staining. (**d**) Samples were prepared as described in (**c**) using 0.5 µM of sHsps. MDH refolding from aggregated or sHsp-complexed states was initiated at 30°C by addition of the *S. saccharomyces* bichaperone system (2 µM Ssa1, 1 µM Sis1, 0.1 µM Sse1, 1 µM Hsp104) and 1 µM GroEL/GroES. MDH activities were determined at the indicated time points. The enzymatic activity of native MDH was set at 100 %. Mean and standard deviation of three independent experiments are shown.

# Supplementary Figure 11

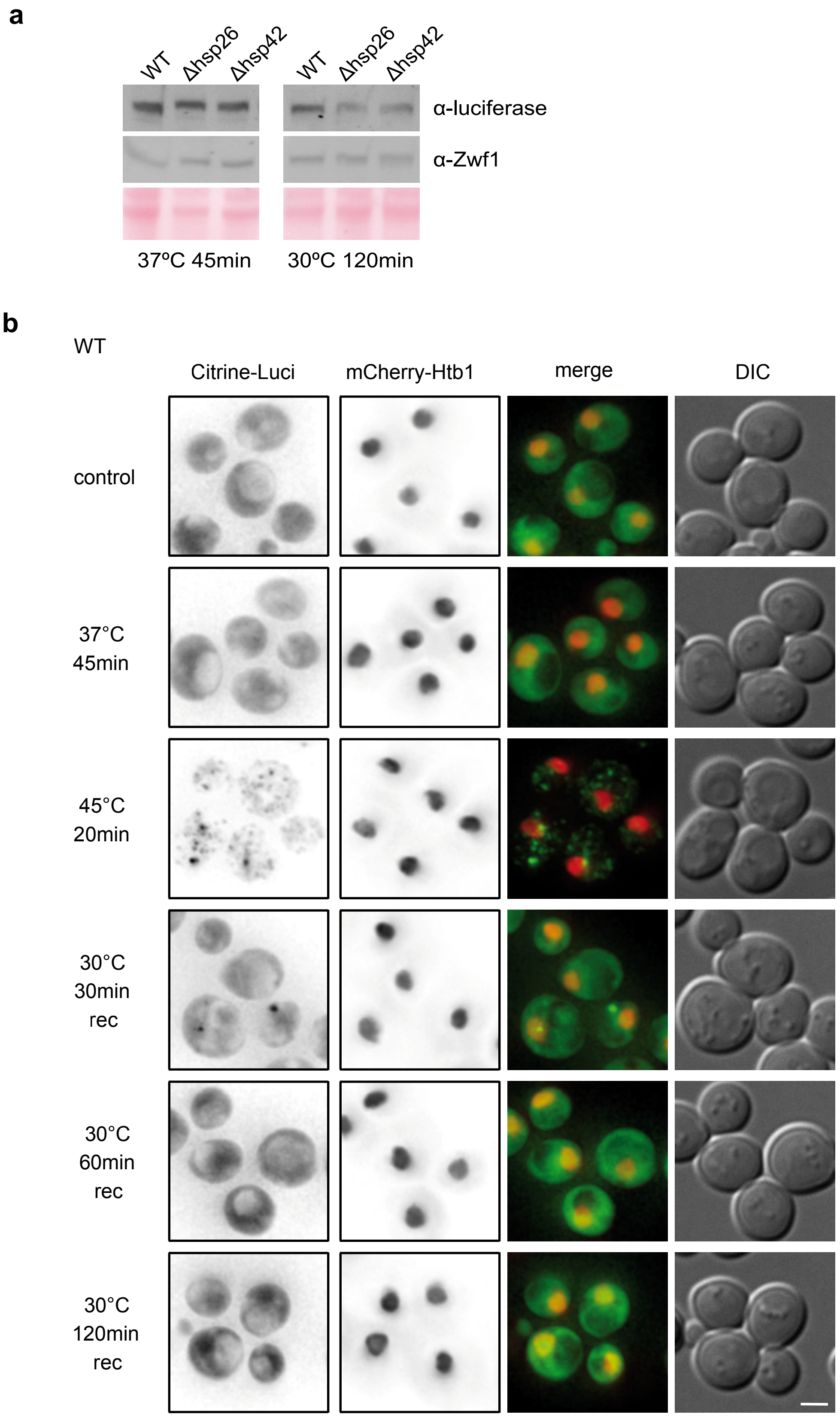

**Supplementary Figure 11**

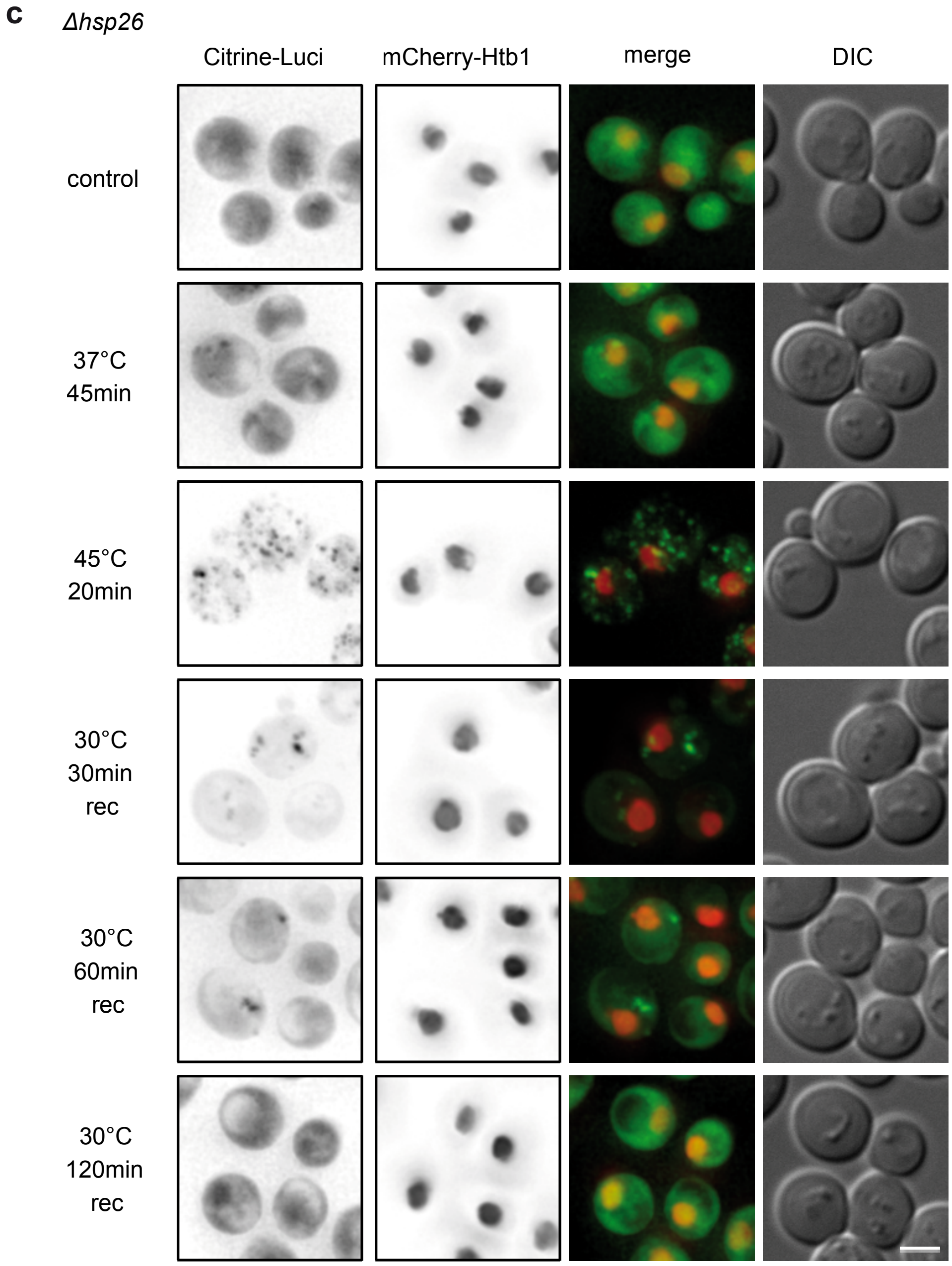

**Supplementary Figure 11**

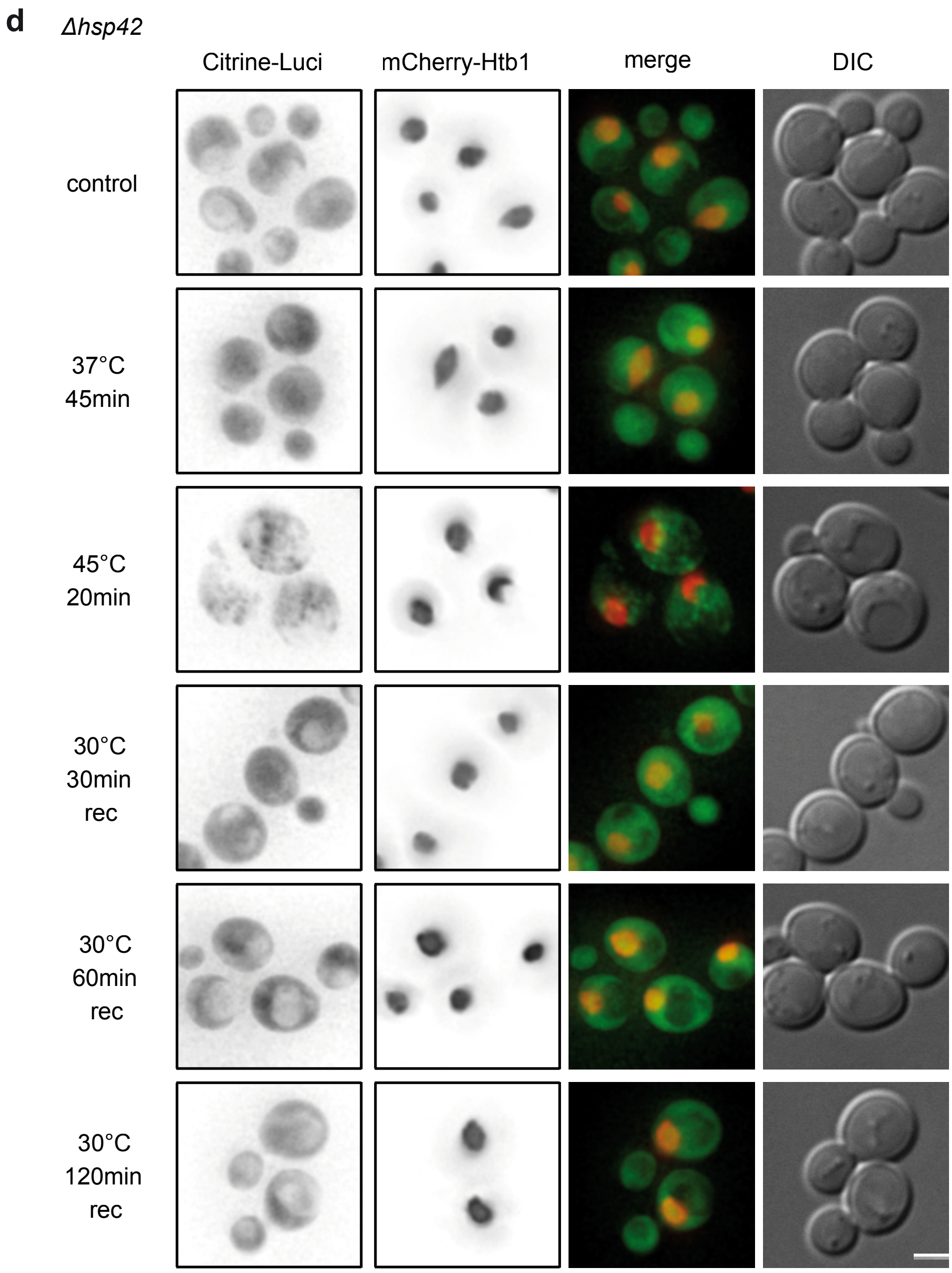


**Supplementary Figure 11**

Aggregation and solubilization of mCitrine-Luciferase. (**a**) mCitrine-luciferase levels prior and post heat shock were determined in *S. cerevisiae* in wt, *hsp26*Δ and *hsp42*Δ cells using Luciferase-specific antibodies. Levels of Zwf1 and the respective section of a Ponceaus S-stained membrane are given as loading control. Uncropped blots are shown in Supplementary Fig. 13. (**b-d**) Heat-induced mCitrine-luciferase aggregates are dissolved more slowly in *Δhsp26* strain (**c**) than in wild type (**b**) or *Δhsp42* (**d**) strains. Localization patterns of mCitrine-luciferase and its superposition with the Htb1 nuclear marker (mCherry) are shown under indicated conditions. Wide-field images are presented and respective DIC images are given. Scale bar 2 μm.

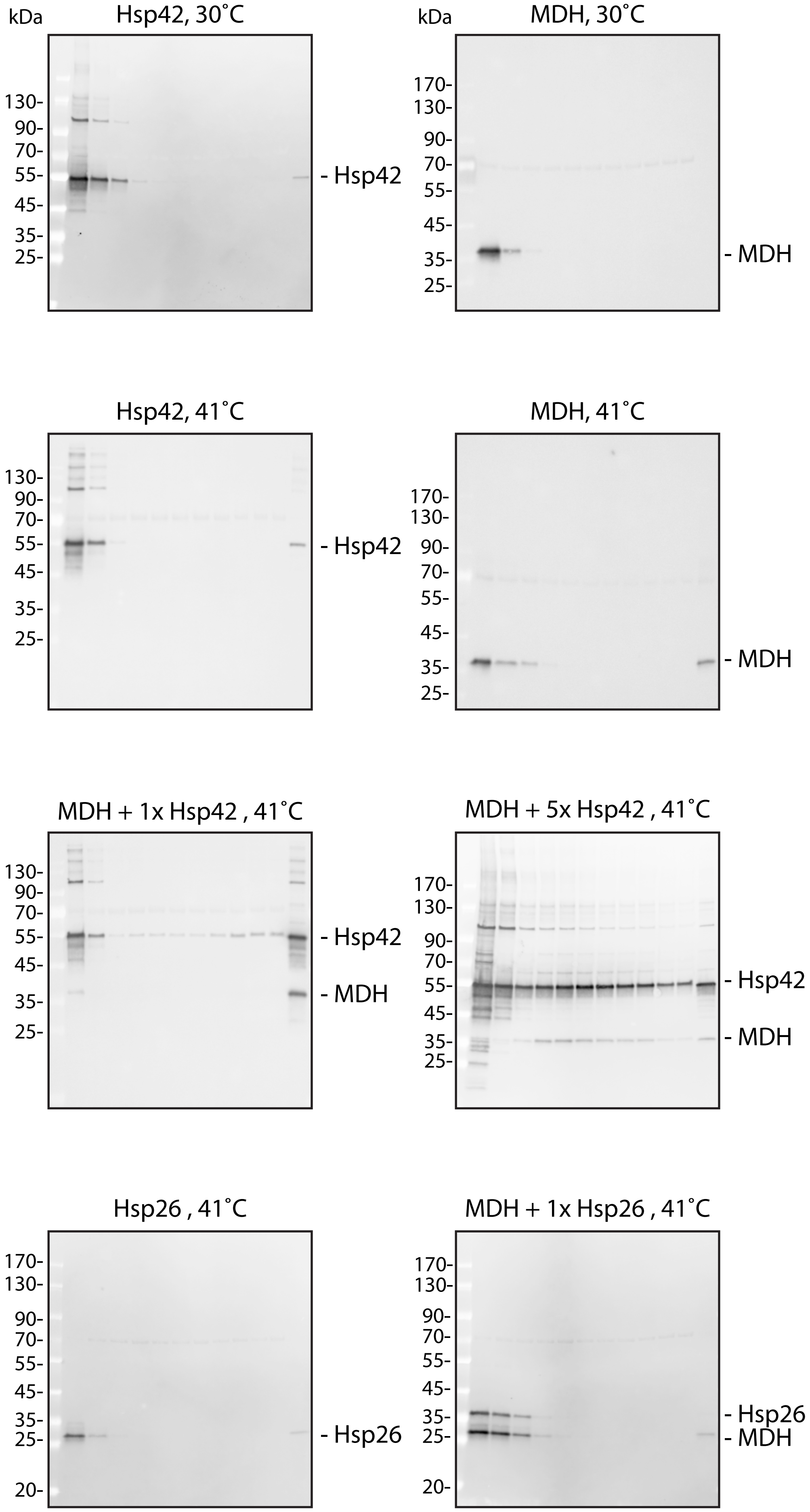


**Supplementary Figure 12**

Hsp42 promotes MDH aggregation under mild denaturation conditions. MDH (0.5 µM) was denatured for 60 min at 41°C in the absence or presence of sHsps at various ratios (0.1 – 2.5 µM). As control 2.5 µM sHsps were heated alone. Samples were loaded onto a 10-50% glycerol gradient and ultracentrifuged at 40 000 rpm for 1 h at 4°C. Fractions were analyzed by SDS-PAGE followed by western blot analysis. A protein standard is given.

# Supplementary Figure 13

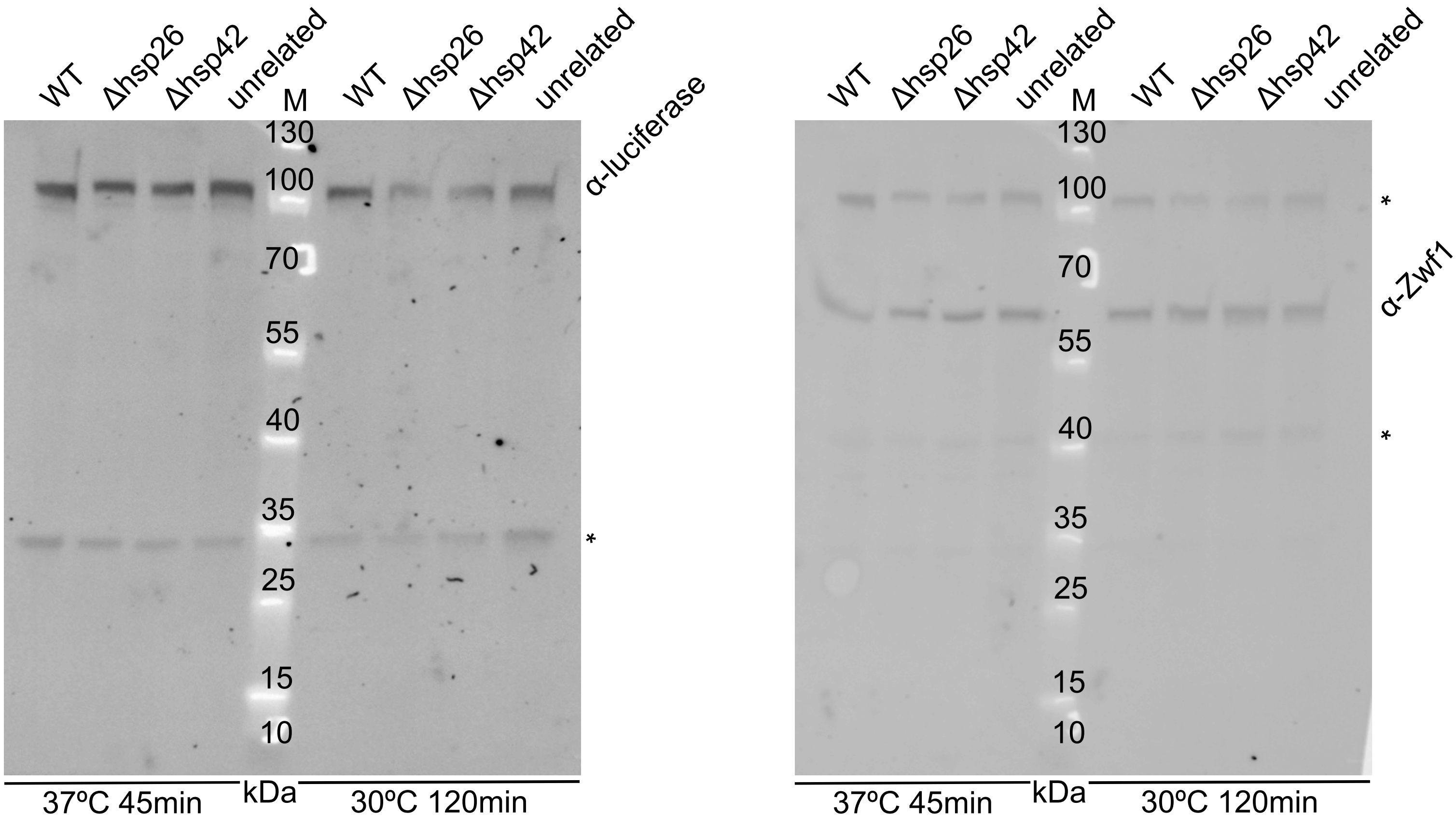


**Supplementary Figure 13**
mCitrine-luciferase levels prior and post heat shock were determined in S. cerevisiae in wt, hsp26Δ and hsp42Δ cells using Luciferase-specific antibodies. Levels of Zwf1 (GAPDH) are given as loading control. A molecular marker (Thermo Scientific, cat.#26616) is given. "*" indicates crossreactivity of the used antibodies.

**Supplementary Table1**: Crosslink products between MDH and Hsp26 after DSS crosslinking of MDH/Hsp26 complexes were identified by mass spectrometry. Different ratios of MDH:Hsp26 were mixed and heat-induced complexes were formed for 30 min at 47°C. DSS crosslinking and subsequent mass spectrometry analysis were performed to identify MDH-sHsp interaction sites. The ld (linear discriminant)-scores for the crosslink products are indicated. NTE: N-terminal extension, ACD: α-crystallin domain, CTE: C-terminal extension.

| MDH-Hsp26-x-link | MDH/Hsp26 1/0.5 | MDH/Hsp26 1/3 | MDH/Hsp26 1/5 | ld-score MDH/Hsp26 1/0.5 | ld-score MDH/Hsp26 1/3 | ld-score MDH/Hsp26 1/5 | Hsp26 domain |
|---|---|---|---|---|---|---|---|
| K55-K45 | | x | x | | 28.59 | 32.12 | NTE |
| K82-K45 | x | x | x | 28.02 | 32.17 | 31.32 | NTE |
| K134-K45 | x | x | x | 32.06 | 23.79 | 33.44 | NTE |
| K180-K45 | | | x | | | 20.16 | NTE |
| K216-K45 | | x | x | | 20.64 | 29.48 | NTE |
| K218-K45 | | x | x | | 34.29 | 32.56 | NTE |
| K278-K45 | x | x | x | 36.74 | 29.41 | 33.86 | NTE |
| K284-K45 | | x | x | | 39.93 | 29.43 | NTE |
| K291-K45 | x | | | 30.24 | | | NTE |
| K301-K45 | | x | | | 20.97 | | NTE |
| K306-K45 | | x | x | | 35.01 | 36.7 | NTE |
| K312-K45 | x | x | x | 20.76 | 24.53 | 29.11 | NTE |
| K134-K50 | | x | x | | 40.61 | 32.07 | NTE |
| K306-K117 | | | x | | | 39.57 | NTE |
| K134-K151 | | | x | | | 30.19 | ACD |
| K216-K151 | | | x | | | 22.62 | ACD |
| K284-K151 | | x | | | 27.84 | | ACD |
| K306-K151 | | x | | | 23.4 | | ACD |
| K312-K151 | | | x | | | 20.91 | ACD |
| K134-K195 | | | x | | | 27.13 | CTE |
| K142-K195 | x | | | 23.72 | | | CTE |
| K216-K195 | x | | | 20.77 | | | CTE |
| K284-K195 | x | | | 32.42 | | | CTE |
| K306-K195 | x | x | x | 35.24 | 33.72 | 25.92 | CTE |
| K278-K198 | | | x | | | 33.62 | CTE |
| K306-K198 | x | x | x | 35.37 | 38.26 | 47.47 | CTE |
| K312-K198 | | | x | | | 24.5 | CTE |

**Supplementary Table 2**: Yeast strains used in this study.

| Name | Genotype | Reference |
|---|---|---|
| **BY4741** | *his3Δ1, leu2Δ0, met15Δ0, ura3Δ0* | EUROSCARF |
| **SSY75** | BY4741 pRS306-PAct1-yEmCitrine-luci* (Ura) pRS305-PAdh1-HTB1-mCherry (Leu) | this study |
| **SSY76** | BY4741 *hsp26Δ::kanMX4* pRS306-PAct1-yEmCitrine-luci* (Ura) pRS305-PAdh1-HTB1-mCherry (Leu) | this study |
| **SSY77** | BY4741 *hsp42Δ::kanMX4* pRS306-PAct1-yEmCitrine-luci* (Ura) pRS305-PAdh1-HTB1-mCherry (Leu) | this study |
| **SSY272** | BY4741 *hsp26Δ::kanMX4* | EUROSCARF |
| **SSY273** | BY4741 *hsp42Δ::kanMX4* | EUROSCARF |
| **SMY571** | BY4741 *pdr5Δ::nat1MX4* pRS303H-PGpd-yeGFP (Hyg) | this study |
| **SMY575** | BY4741 *pdr5Δ::nat1MX4 hsp42?::KANMX4* pRS303H-PGpd-yeGFP (Hyg) | this study |
| **SMY629** | BY4741 *pdr5Δ::nat1MX4 hsp26?::KANMX6* pRS303H-PGpd-yeGFP (Hyg) | this study |

**Supplementary Table 3:** Definition of 4MBP unfolding events in single molecule experiments

| **Reference** | ***ΔL*** **(nm)** | ***F*** **(pN)** | **Note** |
|---|---|---|---|
| Smaller than one core | $\Delta L < 80$ | $F \leq 35$ | Gradual unfolding transitions at low force without distinct steps are also included. |
| Native-like cores | $80 \leq \Delta L \leq 104$ | $F \leq 35$ | *ΔL* range is 12 nm around the core step-size of 92 nm. *F* range is taken as larger than the native core unfolding force plus three times the standard deviation. |
| Weak misfolds | $\Delta L > 104$ | $F < 65$ | 10 nm is taken as the minimally detectable step-size. |
| | $10 \leq \Delta L \leq 104$ | $35 < F < 65$ | |
| Tight misfolds | - | $F > 65$ | Structures cannot be unfolded below the maximum applied force of 65 pN |

**Δ**L: change in contour length; F: unfolding force
